# Association of Methadone Treatment With Substance-Related Hospital Admissions Among a Population in Canada With a History of Criminal Convictions

**DOI:** 10.1001/jamanetworkopen.2019.0595

**Published:** 2019-03-15

**Authors:** Angela Russolillo, Akm Moniruzzaman, Julian M. Somers

**Affiliations:** 1 Somers Research Group, Faculty of Health Sciences, Simon Fraser University, Burnaby, British Columbia, Canada

## Abstract

**Importance:**

People with criminal histories experience high rates of opioid dependence and are frequent users of acute health care services. It is unclear whether methadone adherence prevents hospitalizations.

**Objective:**

To compare hospital admissions during medicated and nonmedicated methadone periods.

**Design, Setting, and Participants:**

A retrospective cohort study involving linked population-level administrative data among individuals in British Columbia, Canada, with provincial justice contacts (n= 250 884) and who filled a methadone prescription between April 1, 2001, and March 31, 2015. Participants were followed from the date of first dispensed methadone prescription until censoring (date of death, or March 31, 2015). Data analysis was conducted from May 1 to August 31, 2018.

**Exposures:**

Methadone treatment was divided into medicated (methadone was dispensed) and nonmedicated (methadone was not dispensed) periods and analyzed as a time-varying exposure.

**Main Outcome and Measures:**

Adjusted hazard ratios (aHRs) of acute hospitalizations for any cause and cause-specific (substance use disorder [SUD], non–substance-related mental disorders [NSMDs], and medical diagnoses [MEDs]) were estimated using multivariable Cox proportional hazards regression.

**Results:**

A total of 11 401 people (mean [SD] age, 34.9 [9.4] years; 8230 [72.2%] men) met inclusion criteria and were followed up for a total of 69 279.3 person-years. During a median follow-up time of 5.5 years (interquartile range, 2.8-9.1 years), there were 19 160 acute hospital admissions. Dispensed methadone was associated with a 50% lower rate of hospitalization for any cause (aHR, 0.50; 95% CI, 0.46-0.53) during the first 2 years (≤2.0 years) following methadone initiation, demonstrating significantly lower rates of admission for SUD (aHR, 0.32; 95% CI, 0.27-0.38), NSMD (aHR, 0.41; 95% CI, 0.34-0.50), and MED (aHR, 0.57; 95% CI, 0.52-0.62). As duration of time increased (2.1 to ≤5.0 years; 5.1 to ≤10.0 years), methadone was associated with a significant but smaller magnitude of effect: SUD (aHR, 0.43; 95% CI, 0.36-0.52; aHR, 0.47; 95% CI, 0.37-0.61), NSMD (aHR, 0.51; 95% CI, 0.41-0.64; aHR, 0.60; 95% CI, 0.47-0.78), and MED (aHR, 0.71; 95% CI, 0.65-0.77; aHR, 0.85; 95% CI, 0.76-0.95).

**Conclusions and Relevance:**

In this study, methadone was associated with a lower rate of hospitalization among a large cohort of Canadian individuals with histories of convictions and prevalent concurrent health and social needs. Practices to improve methadone adherence are warranted.

## Key Points

**Question:**

Is adherence to methadone associated with a lower risk of hospital admission among individuals with criminal convictions?

**Findings:**

In this cohort study including 11 401 Canadian individuals, periods during which methadone was dispensed were associated with lower rates of any acute hospital admission.

**Meaning:**

Adherence to methadone among individuals with criminal convictions may contribute to lower rates of acute hospitalization.

## Teaser

This cohort study compares the risk of substance-related acute hospital admission during periods of methadone-medicated vs nonmedicated periods in individuals in Canada with a history of criminal conviction.

## Introduction

Rising rates of opioid misuse and dependence contribute directly to mortality^1,2^ and disability.^3^ Substance use disorders (SUDs) are overrepresented in correctional settings and among people with criminal histories.^4^ More than half of individuals involved with the justice system report heroin or other drug use prior to incarceration^5^ concurrent with endemic psychiatric disorders and infectious diseases.^6,7,8^ Opioid and other SUDs are often accompanied by complex health needs^9^ and overwhelm acute health care services.^10^ Opioids in particular are associated with hospitalizations^11,12^ and emergency department use^13^ for general as well as offender populations. Canadian surveillance indicates that overdose-related hospitalizations rose 53% over the past decade,^14^ with comparable increases internationally.^15^ In addition, medical issues (eg, soft-tissue infections, infectious diseases) secondary to injection drug use and other risky behaviors place demands on acute care.^16,17,18^ Once hospitalized, patients using opioids have longer lengths of stay^19^ and higher rates of readmission,^20^ with high related costs.^21^ Economic analyses concerning people who are opioid dependent reveal frequent criminal involvement,^22^ poor health status,^6^ and a reliance on hospital services, resulting in a societal cost in excess of $4 billion ($CDN) per year in Canada.^23^ Almost all incarcerated individuals are eventually released to the community, but few inmates with opioid use disorder are released to evidence-based interventions, which remain underused in prisons^24,25^ and communities.^26,27^

A body of research supports the use of methadone maintenance treatment (MMT) to reduce harms associated with opioid use disorder.^28^ Engagement in MMT is associated with reduced mortality^29^ and criminal activity,^30^ as well as lower health care costs.^31,32^ In a systematic review,^33^ MMT was associated with more health benefits and less cost than no drug treatment and was more cost-effective than other agonist treatment options. Although reduced costs may signify diversion from acute care, few studies have examined the association between methadone adherence and hospitalization, and, to our knowledge, none are specific to people with criminal convictions.

In a recent prospective cohort study comparing implanted naltrexone with methadone and buprenorphine, methadone treatment participation was associated with significantly lower rates of hospital admissions and emergency department use.^34^ The study found that receipt of methadone with carry privileges was associated with significantly lower odds of hospital admission,^35^ although this method of dispensing is relatively uncommon. Other studies have reported an association between methadone treatment engagement and lower rates of hospitalization based on relatively small sample sizes^36^ and short follow-up periods.^37^

We investigated the association between dispensed methadone and hospitalization in the population with histories of criminal convictions in British Columbia, Canada, over a 14-year period. We aimed to provide clinical and epidemiologic details of the population and address 2 primary questions related to methadone: Is the risk of hospital admission lower during periods of dispensed methadone compared with nondispensed methadone periods? Is the risk of substance-related hospital admission lower during periods of dispensed methadone relative to other reasons for admission (eg, psychiatric or medical)?

## Methods

### Participants and Data Source

Data were obtained by linking population-level administrative records in British Columbia under the Inter-Ministry Research Initiative. The study cohort consisted of all individuals with histories of provincial justice contacts (n = 250 884) in British Columbia. Those who filled a methadone prescription between April 1, 2001, and March 31, 2015, were eligible for inclusion. We used several population-level databases: the Ministry of Health’s PharmaNet and Discharge Abstract databases and the Ministry of Justice’s registry of convictions. Data were analyzed from May 1 to August 31, 2018. Databases and variables used in this study are listed in eMethods 1, eTable 1, and eTable 2 in the Supplement. Additional details of the Inter-Ministry Research Initiative that are not essential to the present study have been described elsewhere.^38^ The Inter-Ministry Research Initiative serves as a resource for the development of policies and services that span health, justice, and social welfare sectors.

Follow-up extended from the date of first dispensed methadone prescription until censoring (date of death or March 31, 2015). Methadone prescription transactions were collected by the Ministry of Health. Corrections and sociodemographic variables (age, sex, ethnicity, and educational level) were collected by the Ministry of Justice. Ethnicity of participants was ascribed by personnel in the Ministry of Justice, and these categories were used by the study investigators. Hospitalization data were obtained from the Ministry of Health’s Discharge Abstract Database, which includes information related to each acute hospital separation. Covariate information concerning medical and laboratory service use was extracted from the Provincial Medical Services Plan database, which details the date, diagnostic code, and cost associated with medical services to citizens in British Columbia, including while serving sentences under provincial corrections. The study used exclusively retrospective deidentified administrative records and consent was not possible. The study was reviewed and approved without need for waiver of informed consent by the Simon Fraser University Research Ethics Board. This study followed the Strengthening the Reporting of Observational Studies in Epidemiology (STROBE) reporting guideline.

### Measures

Data on the main exposure (methadone) were extracted from the PharmaNet database, a provincewide network linking all prescriptions issued by British Columbia pharmacies. This register omits dispensing information during hospitalization or outside the province of British Columbia. Authorized physicians who hold an exemption from Health Canada are permitted to prescribe methadone in British Columbia.^39^ Methadone is dispensed to individuals who meet criteria for opioid dependence as defined by the *Diagnostic and Statistical Manual of Mental Disorders* (Fifth Edition), and/or *Diagnostic and Statistical Manual of Mental Disorders *(Fourth Edition, Text Revision). People receiving methadone are required to comply with daily witnessed ingestion under the supervision of a pharmacist (ie, attend pharmacy daily to receive dispensed dose of methadone) unless authorized to hold carry privileges.^39^

Methadone was treated as a time-varying exposure (ie, medication status was not constant throughout follow-up), and each participant’s follow-up was divided into medicated (methadone was dispensed) and nonmedicated (methadone was not dispensed) periods. Following the method used in previous research,^30^ a participant was considered exposed to methadone based on pharmacy fill transaction dates. If a participant filled their methadone prescription consistently (no gap in pharmacy transaction dates), the duration of these consistent fills was treated as a single interval/episode and considered as a medicated period (methadone was dispensed). If there were gaps in pharmacy transaction dates, the interval was considered a nonmedicated period (methadone was not dispensed). Participants were expected to alternate between medicated and nonmedicated periods (further details and illustration are presented in eMethods 2 and eTables 3-6 in the Supplement).

The main outcome was 1 or more acute hospital admissions for any cause during follow-up (excluding interhospital transfers). Hospitalizations were classified by most responsible diagnosis for each patient’s stay in the hospital. The present study used *International Statistical Classification of Diseases and Health Problems, Tenth Revision, Canada* (*ICD-10-CA*) to determine the most responsible diagnosis, a modified version of *ICD-10*, developed by Canadian Institute of Health Information and used across Canada. Using *ICD-10-CA* codes, we subdivided hospital admissions for any cause into 3 categories: SUDs (eg, opioids, alcohol), non–substance-related mental disorders (NSMDs) (eg, schizophrenia, depression), and all other medical diagnoses (MEDs) (eg, HIV, cellulitis, or pneumonia). The *ICD-10-CA* coding algorithms have been available in British Columbia since April 1, 2001. Diagnostic codes related to SUD, NSMD, and MED are reported in eTables 7-11 in the Supplement.

### Statistical Analysis

We used descriptive statistics (counts and proportions for nominal variables) and mean (SD) or median and interquartile range (IQR) for continuous variables to characterize the sample. The dependent variable (outcome) was any acute hospital admission. We examined hospitalizations for any cause followed by cause-specific analyses (SUD, NSMD, or MED). Time at risk started when participants filled their first methadone prescription during the observation period and ended when censoring occurred (date of death or March 31, 2015). Time spent in the hospital was excluded from time at risk (eMethods 2 in the Supplement). We chose time-to-event analysis because our outcome of interest was not only the occurrence of an event (hospitalization) but also when it occurred.

Methadone was our primary independent variable and was analyzed as a time-varying covariate (medicated and nonmedicated periods) in the regression model. Both medicated and nonmedicated time segments for methadone were calculated using pharmacy-dispensing/transaction dates (eMethods 2 in the Supplement). Owing to the recurrent nature of the outcome variable, we used the Anderson-Gill counting process method,^40^ an extension of the Cox proportional hazards regression model,^41^ to estimate the hazard of hospital admission associated with dispensed methadone. During the model-building process, we assessed the proportional hazards regression assumption for methadone and other covariates using Schoenfeld residuals.^42^ We found a violation of the assumption of proportionality of methadone in the univariate Cox proportional hazards regression model and refitted the univariate Cox proportional hazards regression model with the interaction terms between methadone and follow-up time at 3 discrete time points (2.0, 5.0, and 10.0 years). This model provided estimates of hazard ratios (HRs) for methadone in each temporal segment (≤2.0 years [≤730 days], 2.1 to ≤5.0 years [731-1826 days], 5.1 to ≤10.0 years [1827-3652 days], and >10.0 years [3653-5111 days]). The multivariable Cox proportional hazards regression model included methadone and temporal interaction terms as well as controlling variables selected based on their established associations with future hospital admissions^13,19,32,37,43,44,45^: age at enrollment, sex, ethnicity, educational level, calendar year, offenses in the previous year, medical services plan cost in the previous year, SUD-related services in the previous year, severe mental illness (ever), and hospitalizations in the previous year (eTable 12 in the Supplement provides a description of study variables).

As an effect size, we report the adjusted HR (aHR) and 95% CI. We also report the risk difference, calculated using the formula *I*_0_ × (aHR − 1), where *I*_0_ is the unadjusted event rate in the unexposed group. To account for dependencies between events within the same individual, we used the robust variance estimator to estimate SEs for parameters.^46,47^ We chose the conventional α level (2-tailed *P* ≤ .05) to interpret the significance of estimated parameters. People with missing demographic information, including ethnicity and educational level, were included in our analyses under the categories titled unknown ethnicity and unknown educational level.

We conducted several subgroup and sensitivity analyses to examine whether our primary results were altered by differences in cohort selection and varying durations of exposure. Associations were examined across 4 subgroups categorized based on different durations of exposure (very short, short, long, and very long) (eTables 13-16 in the Supplement). In addition, we examined participants who exclusively received methadone (no buprenorphine or buprenorphine-naloxone) during the study (eTable 17 in the Supplement). There is evidence demonstrating an association between injection drug use and skin or soft-tissue infections (eg, cellulitis or abscess).^48,49^ To investigate this outcome in the present sample, we conducted additional cause-specific analyses for hospitalizations related to the most responsible diagnoses of cellulitis or abscess (eTable 18 in the Supplement).

## Results

The sample comprised 11 401 people (Figure) (mean [SD] age, 34.9 [9.4] years; 8230 [72.2%] men) who, from April 1, 2001, to March 31, 2015, were followed up for a total of 69 279.3 person-years (PYs). Baseline sociodemographic, criminologic, and hospitalization details for the eligible sample are presented in Table 1. During a median follow-up time of 5.5 years (IQR, 2.8-9.1 years), there were 19 160 acute hospital admissions—a mean (SD) of nearly 2 admissions (1.7 [3.5]) per person or a rate of 27.8 admissions per 100 PYs. A total of 5454 participants (47.8%) had at least 1 acute admission during follow-up. Medical diagnoses accounted for the highest frequency of admissions to hospital (13 273), followed by those related to SUD (1541) and NSMD (1004). The median (IQR) number of medicated and nonmedicated treatment years (1.6 [0.4-3.9] and 2.5 [0.7-5.2] years, respectively) represented a total medicated treatment time of 29 706.8 PYs and a nonmedicated treatment time of 39 572.4 PYs. A total of 155 participants (1.4%) were dispensed methadone for the entire observation period (no nonmedicated periods), and 971 participants received buprenorphine or buprenorphine-naloxone in the follow-up period. Only a single participant received buprenorphine, and all others received buprenorphine-naloxone.

**Figure.**
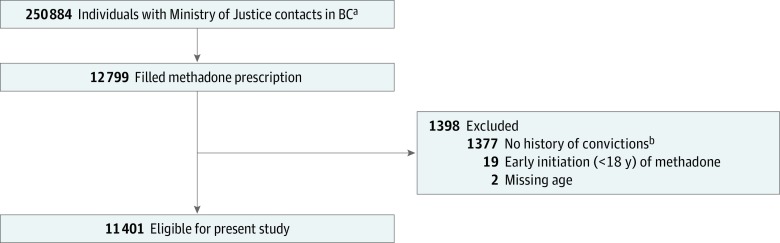
Flowchart of Patients With Convictions in British Columbia (BC), 2001-2015, Who Were Receiving Methadone ^a^The cohort included participants (offenders) who had convictions (found or pleaded guilty and sentenced) as well as those (nonoffenders) who did not have any convictions but were under supervision of the Ministry of Justice due to remand or bail and later found not guilty (nonoffenders). ^b^This time period included the study/exposure period (April 1, 2001, to March 31, 2015) for methadone as well as the time before enrollment (from the time when justice databases became available in January 1997).

**Table 1.** Sociodemographic, Methadone, and Crime-Related Characteristics of 11 401 Patients With Convictions in British Columbia, 2001-2015, Who Were Receiving Methadone

Variable	No. (%)
Age at enrollment, y^a^	
Mean (SD)	34.9 (9.4)
Median (IQR) [range]	33.6 (27.3-41.5) [18.0-74.9]
Age groups, y	
18 to < 25	1812 (15.9)
25 to < 35	4431 (38.9)
35 to < 45	3296 (28.9)
45 to < 55	1562 (13.7)
≥55	300 (2.6)
Men	8230 (72.2)
Ethnicity	
White	8196 (71.9)
Indigenous	1758 (15.4)
Other	993 (8.7)
Unknown	454 (4.0)
Educational level	
<Grade 10	1483 (13.0)
Grade 10 or 11	3926 (34.4)
Grade 12	3765 (33.0)
Vocational or university	1303 (11.4)
Unknown	924 (8.1)
Follow-up period, y^b^	
Mean (SD)	6.1 (4.0)
Median (IQR) [range]	5.5 (2.8-9.1) [<0.1-14.0]
Total follow-up time, PYs	69 279.3
Hospital length of stay during follow-up period, d	
Mean (SD)	14.7 (42.0)
Median (IQR) [range]	0 (0-9) [0-928]
Total hospital stay, y	459.5
Time at risk, y^c^	
Mean (SD)	6.0 (3.9)
Median (IQR) [range]	5.4 (2.8-9.0) [<0.1-14.0]
Total time at risk, y	68 819.8
Year of methadone initiation	
2001-2005	3026 (26.5)
2006-2010	4313 (37.8)
2011-2015^d^	4062 (35.6)
Medicated period, y	
Mean (SD)	2.6 (2.8)
Median (IQR) [range]	1.6 (0.4-3.9) [<0.1-13.8]
Total medicated time, PYs	29 706.8
No. of medicated periods or episodes	
Mean (SD)	37.3 (49.9)
Median (IQR) [range]	19 (6-49) [1-501]
Nonmedicated period, y	
Mean (SD)	3.5 (4.0)
Median (IQR) [range]	2.5 (0.7-5.2) [0.0-14.0]
Total nonmedicated time, PYs	39 572.4
No. of nonmedicated periods or episodes^e^	
Mean (SD)	36.9 (49.8)
Median (IQR) [range]	19 (5-48) [0-501]
No. of methadone transactions in the year after enrollment (n = 10 376), mean (SD)^f^	166.9 (120.6)
Received buprenorphine or buprenorphine-naloxone in follow-up period	971 (8.5)^g^
Pharmacy transactions in the year after enrollment (n = 930), mean (SD)^h^	
No. of buprenorphine or buprenorphine-naloxone transactions	7.8 (29.9)
No. of methadone transactions	152.0 (111.7)
Severe mental illness, No. (%)	
No schizophrenia or bipolar disorder	7374 (64.7)
Schizophrenia	1772 (15.5)
Bipolar disorder	2255 (19.8)
No. of offenses in the year prior to enrollment, mean (SD)	1.0 (2.0)
Any offense in the year prior to enrollment, offenses, No. (%)	
None	7202 (63.2)
1-2	2664 (23.4)
>2	1535 (13.5)
No. of jail sentences in the year prior to enrollment, mean (SD)	0.6 (1.7)
Participants with ≥1 jail sentence in the year prior to enrollment, No. (%)	2175 (19.1)
Jail sentence during follow-up period	
Mean (SD), No.	2.8 (6.2)
Median (IQR) [range], No.	0 (0-3) [0-86]
Total jail sentences, No.	31 498
Rate per PY	0.45
≥1 Jail sentence, No. (%)	4143 (36.3)
MSP cost in the year prior to enrollment, No. (%), $^i^	
First quartile (≤295)^j^	2851 (25.0)
Second quartile (296-751)	2850 (25.0)
Third quartile (752-1743)	2850 (25.0)
Fourth quartile (≥1744)	2850 (25.0)
MSP services (SUD related) in the year prior to enrollment, No. (%)	
Low (≤1)^k^	5841 (51.2)
Medium (2-6)	2957 (25.9)
High (≥7)	2603 (22.8)

Abbreviations: IQR, interquartile range; MSP, medical services plan; PYs, person-years; SUD, substance use disorder.

^a^ Age at enrollment was based on date of initiation of methadone treatment (between April 1, 2001, to March 31, 2015).

^b^ The follow-up period was estimated from the time difference between time 0 (initiation of methadone) and study end (death or March 31, 2015).

^c^ Length of stay during the follow-up period was excluded from the analysis time. Time at risk was calculated subtracting the hospital days from the follow-up period.

^d^ Only 3 months (January to March) of data were included in 2015.

^e^ A total of 155 participants (1.4%) did not have any nonmedicated treatment periods and received methadone during the entire observation period.

^f^ Restricted to participants (n = 10 376) who had at least 1 year of follow-up.

^g^ Only a single participant received buprenorphine; all others received buprenorphine-naloxone.

^h^ Restricted to participants (n = 930) who received buprenorphine or buprenorphine-naloxone and had at least 1 year of follow-up.

^i^ All costs in Canadian dollars.

^j^ The 25th, 50th, and 75th percentiles were used to categorize MSP costs into 4 separate groups.

^k^ The 50th and 75th percentiles were used to categorize the groups as low, medium, and high.

For hospital admissions due to any cause (Table 2), dispensed methadone was associated with a 50% lower rate of hospitalization (aHR, 0.50; 95% CI, 0.46-0.53) during the first 2 years (≤2.0 years) following methadone initiation, equating to a risk difference of 20.7 fewer hospital admissions per 100 PYs. Moreover, dispensed methadone was associated with a lower rate of admission for SUD (aHR, 0.32; 95% CI, 0.27-0.38), NSMD (aHR, 0.41; 95% CI, 0.34-0.50), and MED (aHR, 0.57; 95% CI, 0.52-0.62) during the first 2 years of treatment (Table 3). As duration of time following methadone initiation increased (2.1 to ≤5.0 years; 5.1 to ≤10.0 years), smaller but statistically significant associations between dispensed methadone were observed for admissions related to SUD (2.1 to ≤5.0 years: aHR, 0.43; 95% CI, 0.36-0.52; 5.1 to ≤10.0 years: aHR, 0.47; 95% CI, 0.37-0.61), NSMD (2.1 to ≤5.0 years: aHR, 0.51; 95% CI, 0.41-0.64; 5.1 to ≤10.0 years; aHR, 0.60; 95% CI, 0.47-0.78), and MED (2.1 to ≤5.0 years: aHR, 0.71; 95% CI, 0.65-0.77; 5.1 to ≤10.0 years: aHR, 0.85; 95% CI, 0.76-0.95).

**Table 2.** Extended Cox Proportional Hazards Regression Analysis Estimating the Hazards Associated With Methadone for Any-Cause Acute Hospitalizations Among 11 401 Patients With Convictions in British Columbia, 2001-2015, Who Were Receiving Methadone

Time Segment, y^a^	Period Receiving Methadone	Total Admissions, No.	Total PYs	Incidence per 100 PYs	HR (95% CI)		Risk Difference per 100 PYs (95% CI)^e^
					Unadjusted^b^^,^^c^	Adjusted^d^	
≤2.0	No	4129	9984.5	41.4	1 [Reference]	1 [Reference]	
	Yes	2416	10 612.5	22.8	0.52 (0.48 to 0.56)	0.50 (0.46 to 0.53)	−20.7 (−22.3 to −19.4)
2.1 to ≤5.0	No	4485	14 043.6	31.9	1 [Reference]	1 [Reference]	
	Yes	2057	9206.6	22.3	0.70 (0.65 to 0.76)	0.63 (0.59 to 0.69)	−11.8 (−13.1 to −9.9)
5.1 to ≤10.0	No	3228	12 020.6	26.9	1 [Reference]	1 [Reference]	
	Yes	1746	7889.5	22.1	0.82 (0.75 to 0.91)	0.75 (0.68 to 0.83)	−6.7 (−8.6 to −4.6)
>10.0	No	646	3101.8	20.8	1 [Reference]	1 [Reference]	
	Yes	453	1960.7	23.1	1.11 (0.92 to 1.34)	1.00 (0.83 to 1.21)	0.0 (−3.5 to 4.4)
Overall	No	12 488	39 150.5	31.9	NA	NA	NA
	Yes	6672	29 669.3	22.5			
	Total	19 160	68 819.8	27.8			

Abbreviations: HR, hazard ratio; NA, not applicable; PYs, person-years.

^a^ The ranges of days for the 4 time segments are as follows: 730 days or less (≤2.0 years), 731 to 1826 days (2.1 to ≤5.0 years), 1827 to 3652 days (5.1 to ≤10.0 years), and 3653 to 5111 days (>10.0 years).

^b^ This Cox proportional hazards regression model includes treatment and the interaction terms with time segments at 2.0, 5.0, and 10.0 years.

^c^ The 95% CIs and both unadjusted and adjusted HRs were estimated using robust SEs.

^d^ The multivariable Cox proportional hazards regression model was controlled for age at enrollment (18 to <25, 25 to <35, 35 to <45, 45 to <55, and ≥55 years), sex (men and women), ethnicity (white, indigenous, other, and unknown), educational level (less than grade 10, grade 10 or 11, grade 12, vocational or university, and unknown), calendar year (2001-2005, 2006-2010, and 2011-2015), offenses in the previous year (none, 1-2, and ≥3 offenses), medical services plan cost in the previous year (quartile variable), substance use disorder–related services in the previous year (0-1, 2-6, and ≥7 services), severe mental illness (no schizophrenia or bipolar disorder, schizophrenia and bipolar disorder), and hospitalizations in the previous year (no vs yes).

^e^ Risk difference was calculated using the formula: *I*_0_ × (adjusted HR − 1), where I_0_ indicates unadjusted event rate in the unexposed group (nonmedicated).

**Table 3.** Extended Cox Proportional Hazards Regression Analysis Estimating the Hazards Associated With Methadone for SUD-, NSMD-, and MED-Related Hospitalizations Among 11 401 Patients With Convictions in British Columbia, 2001-2015, Who Were Receiving Methadone ^a^^,^^b^^,^^c^

Time Segment, y^d^	Period Receiving Methadone	Total Admissions	Total PYs	Incidence per 100 PYs	HR (95% CI)		Risk Difference per 100 PYs (95% CI)^h^
					Unadjusted^e^^,^^f^	Adjusted^g^	
**SUD**							
≤2.0	No	850	9984.5	8.5	1 [Reference]	1 [Reference]	
	Yes	309	10 612.5	2.9	0.33 (0.28 to 0.38)	0.32 (0.27 to 0.38)	−5.8 (−6.2 to −5.3)
2.1 to ≤5.0	No	821	14 043.6	5.8	1 [Reference]	1 [Reference]	
	Yes	268	9206.6	2.9	0.50 (0.41 to 0.60)	0.43 (0.36 to 0.52)	−3.3 (−3.7 to −2.8)
5.1 to ≤10.0	No	586	12 020.6	4.9	1 [Reference]	1 [Reference]	
	Yes	214	7889.5	2.7	0.56 (0.44 to 0.71)	0.47 (0.37 to 0.61)	−2.6 (−3.1 to −1.9)
>10.0	No	100	3101.8	3.2	1 [Reference]	1 [Reference]	
	Yes	70	1960.7	2.9	0.90 (0.58 to 1.39)	0.76 (0.49 to 1.16)	−0.8 (−1.6 to 0.5)
Overall	No	2357	39 150.5	6.0	NA	NA	NA
	Yes	848	29 669.3	2.9			
	Total	3205^i^	68 819.8	4.7			
**NSMD**							
≤2.0	No	549	9984.5	5.5	1 [Reference]	1 [Reference]	
	Yes	270	10 612.5	2.5	0.44 (0.36 to 0.53)	0.41 (0.34 to 0.50)	−3.2 (−3.6 to −2.7)
2.1 to ≤5.0	No	536	14 043.6	3.8	1 [Reference]	1 [Reference]	
	Yes	222	9206.6	2.4	0.63 (0.51 to 0.79)	0.51 (0.41 to 0.64)	−1.9 (−2.3 to −1.4)
5.1 to ≤10.0	No	369	12 020.6	3.1	1 [Reference]	1 [Reference]	
	Yes	186	7889.5	2.4	0.77 (0.59 to 1.00)	0.60 (0.47 to 0.78)	−1.2 (−1.6 to −0.7)
>10.0	No	70	3101.8	2.3	1 [Reference]	1 [Reference]	
	Yes	30	1960.7	1.5	0.67 (0.42 to 1.07)	0.51 (0.32 to 0.81)	−1.1 (−1.5 to −0.4)
Overall	No	1524	39 150.5	3.9	NA	NA	NA
	Yes	708	29 669.3	2.4			
	Total	2232^j^	68 819.8	3.2			
**MED**							
≤2.0	No	2730	9984.5	27.3	1 [Reference]	1 [Reference]	
	Yes	1837	10 612.5	17.3	0.59 (0.55 to 0.65)	0.57 (0.52 to 0.62)	−11.8 (−13.1 to −10.4)
2.1 to ≤5.0	No	3128	14 043.6	22.3	1 [Reference]	1 [Reference]	
	Yes	1567	9206.6	17.0	0.76 (0.70 to 0.83)	0.71 (0.65 to 0.77)	−6.5 (−7.8 to −5.1)
5.1 to ≤10.0	No	2273	12 020.6	18.9	1 [Reference]	1 [Reference]	
	Yes	1346	7889.5	17.1	0.90 (0.81 to 1.01)	0.85 (0.76 to 0.95)	−2.8 (−4.5 to −0.9)
>10.0	No	476	3101.8	15.3	1 [Reference]	1 [Reference]	
	Yes	366	1 960.7	18.7	1.22 (0.98 to 1.51)	1.15 (0.93 to 1.43)	2.3 (−1.1 to 6.6)
Overall	No	8607	39 150.5	22.0	NA	NA	NA
	Yes	5116	29 669.3	17.2			
	Total	13 273	68 819.8	19.9			

Abbreviations: HR, hazard ratio; MED, medical diagnoses; NA, not applicable; NSMD, non–substance-related mental disorder; PYs, person-years; SUD, substance use disorder.

^a^ Hospitalizations associated with SUD were determined using most responsible (primary) diagnostic codes (F10-F19) for in-hospital stay.

^b^ Hospitalizations associated with NSMD were determined using most responsible (primary) diagnostic codes (F00-F09; F20-F99) for in-hospital stay.

^c^ Nonpsychiatric hospitalizations were determined using most responsible (primary) diagnostic codes for in-hospital stay and included all the residual diagnostic codes excluding codes for NSMD (F00-F09, F20-F99) and SUD (F10-F19).

^d^ The ranges of days for the 4 time segments are as follows: 730 days or less (≤2.0 years), 731 to 1826 days (2.1 to ≤5.0 years), 1827 to 3652 days (5.1 to ≤10.0 years), and 3653 to 5111 days (>10.0 years).

^e^ This Cox proportional hazards regression model includes treatment and the interaction terms with time segments at 2.0, 5.0, and 10.0 years.

^f^ The 95% CIs and both unadjusted and adjusted HRs were estimated using robust SEs.

^g^ The multivariable Cox proportional hazards regression model was controlled for age at enrollment (18 to <25, 25 to <35, 35 to <45, 45 to <55, and ≥55 years); sex (men and women); ethnicity (white, indigenous, other, and unknown), educational level (less than grade 10, grade 10 or 11, grade 12, vocational or university, and unknown), calendar year (2001 to 2005, 2006 to 2010 and 2011 to 2015), offenses in the previous year (none, 1-2, and ≥3 offenses), medical services plan cost in the previous year (quartile variable), SUD-related services in the previous year (0-1, 2-6, and ≥7 services), severe mental illness (no schizophrenia or bipolar disorder, schizophrenia bipolar disorder), and hospitalizations in the previous year (no vs yes).

^h^ Risk difference was calculated using the formula: *I*_0_ × (adjusted HR − 1), where *I*_0_ is the unadjusted event rate in the unexposed group (nonmedicated).

^i^ Represents 16.7% of all hospital admissions (n = 19 160).

^j^ Represents 11.6% of all hospital admissions (n = 19 160).

Dispensed methadone was not associated with significant reductions in hospitalization risk in periods exceeding 10.0 years following methadone initiation with the exception of NSMD-related hospitalizations (aHR, 0.51; 95% CI, 0.32-0.81). After exclusion of participants who died during follow-up (n = 762), the association between dispensed methadone and lower risk of hospitalization for any cause remained significant across all time segments up to 10 years following methadone initiation (≤2.0 years: aHR, 0.50; 95% CI, 0.46-0.54; 2.1 to ≤5.0 years: aHR, 0.62; 95% CI, 0.57-0.68; and 5.1 to ≤10.0 years: aHR, 0.74; 95% CI, 0.66-0.82) (Table 4).

**Table 4.** Subgroup Analysis Estimating the Hazards Associated With Methadone for Any-Cause Acute Hospitalizations Among 10 639 Surviving Patients With Convictions in British Columbia, 2001-2015, Who Were Receiving Methadone^a^

Time Segment, y^b^	Period Receiving Methadone	Total Admissions, No.	Total PYs	Incidence per 100 PYs	HR (95% CI)		Risk Difference per 100 PYs (95% CI)^f^
					Unadjusted^c^^,^^d^	Adjusted^e^	
≤2.0	No	3557	9423.1	37.7	1 [Reference]	1 [Reference]	
	Yes	2061	9973.7	20.7	0.52 (0.48 to 0.56)	0.50 (0.46 to 0.54)	−18.9 (−20.4 to −17.4)
2.1 to ≤5.0	No	3842	13 427.6	28.6	1 [Reference]	1 [Reference]	
	Yes	1713	8699.5	19.7	0.69 (0.63 to 0.75)	0.62 (0.57 to 0.68)	−10.9 (−12.3 to −9.2)
5.1 to ≤10.0	No	2804	11 623.5	24.1	1 [Reference]	1 [Reference]	
	Yes	1486	7581.3	19.6	0.81 (0.73 to 0.90)	0.74 (0.66 to 0.82)	−6.3 (−8.2 to −4.3)
>10.0	No	573	3063.1	18.7	1 [Reference]	1 [Reference]	
	Yes	402	1934.3	20.8	1.11 (0.92 to 1.36)	0.99 (0.82 to 1.21)	−0.2 (−3.4 to 3.9)
Overall	No	10 766	37 537.3	28.7	NA	NA	NA
	Yes	5662	28 188.7	20.1			
	Total	16 438	65 726.0	25.0			

Abbreviations: HR, hazard ratio; NA, not applicable; PYs, person-years.

^a^ Analysis was restricted to participants who did not die during the study period (762 participants [6.7%] died during the study period).

^b^ The ranges of days for the four time segments are as follows: 730 days or less (≤2.0 years), 731 to 1826 days (2.1 to ≤5.0 years), 1827 to 3652 days (5.1 to ≤10.0 years), and 3653 to 5111 days (>10.0 years).

^c^ This Cox proportional hazards regression model includes treatment and the interaction terms with time segments at 2.0, 5.0, and 10.0 years.

^d^ The 95% CIs and both unadjusted and adjusted HRs were estimated using robust SEs.

^e^ The multivariable Cox proportional hazards regression model was controlled for age at enrollment (18 to <25, 25 to <35, 35 to <45, 45 to <55, and ≥55 years), sex (men and women), ethnicity (white, indigenous, other, and unknown), educational level (less than grade 10, grade 10 or 11, grade 12, vocational or university, and unknown), calendar year (2001-2005, 2006-2010, and 2011-2015), offenses in the previous year (none, 1-2, and ≥3 offenses), medical services plan cost in the previous year (quartile variable), substance use disorder–related services in the previous year (0-1, 2-6, and ≥7 services), severe mental illness (no schizophrenia or bipolar disorder, schizophrenia and bipolar disorder), and hospitalizations in the previous year (no vs yes).

^f^ Risk difference was calculated using the formula: *I*_0_ × (adjusted HR − 1), where *I*_0_ indicates unadjusted event rate in the unexposed group (nonmedicated).

### Sensitivity and Subgroup Analyses

The association between hospitalization and dispensed methadone was further investigated in subgroup and sensitivity analyses (eTables 13-18 in the Supplement). After repeating our analyses using varying durations of exposure (≤2.0 years [very short]; 2.1 to ≤5.0 years [short]; and 5.1 to ≤10.0 years [long]), the risk of acute hospitalization was lower during periods of dispensed methadone in each instance (eTables 13-15 in the Supplement). When restricted to those with at least 10 years of follow-up (n = 2322), the association between dispensed methadone and hospitalizations for any cause was attenuated across very long methadone treatment periods (>10.0 years) (eTable 16 in the Supplement). In time periods up to and including 10 years, methadone treatment was associated with a significantly lower risk of hospitalization (≤2.0 years: aHR 0.49; 95% CI, 0.46-0.53; 2.1 to ≤5.0 years: 0.64; 95% CI, 0.59-0.70; and 5.1 to ≤10.0 years: 0.76; 95% CI, 0.69-0.85) (eTable 17 in the Supplement) among participants with methadone monotherapy (n=10 430) and no other forms of opioid agonist treatment (buprenorphine or buprenorphine-naloxone) and did not differ from our main results. In sensitivity analyses, we separately examined hospitalizations related to specific MEDs (abscess and cellulitis) (eTable 18 in the Supplement), which supported the association between dispensed methadone and lower risk of hospital admission across shorter follow-up periods (≤2.0 years; 2.1 to ≤5.0 years) but diminished in periods exceeding 5 years.

## Discussion

Dispensed methadone was associated with a significantly lower risk of hospitalization for any cause among individuals with opioid dependence and histories of criminal convictions. To our knowledge, this is the first study to investigate the association between methadone adherence and hospital admissions in a large sample (>10 000) over periods exceeding 10 years.

We found that dispensed methadone was associated with a significantly lower risk of hospitalization for SUD, NSMD, and MED, and that among these conditions, the greatest magnitude of association was with SUD. The health status of people in our sample is consistent with prior epidemiologic findings involving individuals with criminal histories, confirming significant health inequities^50^ and multiple comorbidities necessitating acute intervention.^51,52^ Our results demonstrate substantial risk for hospitalizations related to psychiatric and other medical issues when individuals were not receiving methadone. This outcome is expected given the evidence that health service use is highest among offenders with co-occurring substance use and mental illness in comparison with either disorder alone.^53,54^ The opportunity to reduce the risk of acute hospital admissions via methadone should be considered in combination with recovery-oriented frameworks^55^ and long-term care needs^56^ targeting individuals with co-occurring disorders.^57^ In addition, we observed a statistically significant association between any acute admission and methadone during the first 2 years following treatment initiation and extending beyond a decade for psychiatric-related hospitalizations. Our findings support calls for integrated^58,59^ and inclusive^60^ health care (eg, opioid use, psychiatric disorders), pharmacotherapy, and non–health-related services (eg, housing, psychosocial support) in a patient-centered and low barrier approach.^61^

Nearly all incarcerated individuals return to the community and face distinct health and social risks during this transition.^62^ Continuity of care is often compromised, and discrimination contributes to suboptimal substance use treatment engagement,^63^ promoting a reliance on acute hospital services.^64^ This vulnerable period has signaled calls for partnerships between health and correctional institutions to minimize exposure to unnecessary risks for both individual offenders and the communities involved.^65^ Our results indicate that many medical-related hospital admissions were indirect consequences of opioid use (eg, cellulitis, HIV) and are preventable. Reviewers have identified a substantial gap between policy^66^ and evidence-based approaches^25,67^ for people who have been involved with the justice system.^68^ In attempts to bridge this gap, studies have evaluated the importance of early engagement in primary care^69^ and team-based behavioral health models in the postrelease period,^70^ demonstrating significant reductions in emergency department use and readmission rates, respectively. In light of the known harms of drug criminalization and the current opioid crisis, it has been argued that there is a moral imperative to improve the standard of substance use disorder care available to people under correctional supervision.^71^

### Strengths and Limitations

Our study has several strengths, including its large population-based sample with nearly 2 decades of comprehensive hospitalization and pharmacologic data. We also have limitations to consider. The use of a Canadian population with correctional histories and health services that are publicly funded may limit the generalizability of our results to other settings and jurisdictions. Our reliance on administrative data is subject to bias related to missing or incomplete records and errors associated with prescriber judgment (eg, not initiating MMT despite being indicated). Our outcome was restricted to admissions recorded in the provincial hospital database, and therefore it failed to account for hospital admissions outside of British Columbia. Receipt of methadone treatment is often accompanied by psychosocial supports, such as counseling, psychological treatments (eg, motivational interviewing, cognitive behavioral therapy, or relapse prevention), Alcoholics Anonymous, and Narcotics Anonymous, with varying degrees of access and participation among individuals. Involvement with ancillary supports was not accounted for in our analyses and may have altered hospitalization risk independent of MMT.

Methadone prescribing in British Columbia almost universally involves witnessed methadone ingestion, and therefore our use of pharmacy dispensing records provides a basis for inferring methadone adherence; however, we are unable to account for the potential diversion of methadone following administration. Disruptions to treatment, such as relocation, were not assessed and may have influenced the integrity of our results. Methadone dose was not accounted for in our analysis, and although a dose-response relationship has been established between methadone and other treatment outcomes (eg, retention), there is no clear evidence that dose is associated with hospital admission.^35^

## Conclusions

Our study shows an association of opioid use with high rates of hospital admissions and demonstrates an association between methadone adherence and the likelihood of admission. Given the complex physical and mental health needs of most opioid-dependent people, policies should support engagement of those who are marginalized and at high risk for both hospitalization and justice system involvement to improve methadone adherence and overall recovery.

## Notes

Supplement. **eMethods 1.** Description of Databases Used in the Study **eTable 1.** Summary of Information Available From the Ministry Of Health Database Used in the Current Study **eTable 2.** Summary of Information Available From the Ministry of Justice Database Used in the Current Study **eMethods 2.** Methods for Constructing Time Intervals and Time at Risk in the Cox Regression Model **eTable 3.** Illustration of Data Structure for the Primary Exposure Variable (Methadone) in the Cox Regression for Five Hypothetical Participants **eTable 4.** Illustration of Event Time (Admission & Discharge) for Five Hypothetical Participants **eTable 5.** Illustration of the Data Structure for the Primary Exposure and Outcome Variables in the Cox Regression for Five Hypothetical Participants **eTable 6.** Illustration of Summary Statistics for Five Hypothetical Participants **eTable 7.** Top 5 ICD-10-CA Diagnostic Categories Responsible for SUD, NSMD, and MED related acute hospitalizations among 11,401 Methadone Patients With Convictions From BC, 2001–2015 **eTable 8.** ICD-10 Diagnostic Codes (F10-F19) for SUD Related Acute Hospitalizations (n=3,205) **eTable 9.** ICD-10-CA Diagnostic Codes (F00-F09; F20-F99) for NSMD Related Acute Hospitalizations (n=2,232) **eTable 10.** ICD-10 Diagnostic Codes for MED Acute Hospitalizations (N = 13 723) **eTable 11.** ICD-9 Diagnostic CODES (290-319) for Mental Disorders **eTable 12.** Description of Methadone and Controlling Variables Included in the Multivariable Cox Regression **eTable 13.** Subgroup Analysis Estimating the Hazard Associated With Methadone for Acute Hospitalizations (Any Cause) Among 1,978 Methadone Patients With Convictions and Very Short Follow Up (<2 Years) From BC, 2001-2015 **eTable 14.** Subgroup Analysis Estimating the Hazard Associated With Methadone for Acute Hospitalizations (Any Cause) Among 3,248 Methadone Patients With Convictions and Short Follow Up (2-5 Years) From BC, 2001-2015 **eTable 15.** Subgroup Analysis Estimating the Hazard Associated With Methadone for Acute Hospitalizations (Any Cause) Among 3,853 Methadone Patients With Convictions And Long Follow Up (5-10 Years) From BC, 2001-2015 **eTable 16.** Subgroup Analysis Estimating the Hazard Associated With Methadone for Acute Hospitalizations (Any Cause) Among 2,322 Methadone Patients With Convictions and Very Long Follow Up (>10 Years) From BC, 2001-2015 **eTable 17.** Subgroup Analysis Estimating the Hazard Associated With Methadone for Acute Hospitalizations (Any Cause) Among 10,430 Methadone Patients With Convictions Only Prescribed Methadone (No Buprenorphine or Buprenorphine-Naloxone) From BC, 2001-2015 **eTable 18.** Extended Cox Regression Analysis Estimating the Hazard Associated With Methadone for Cellulitis or Abscess Related Hospitalizations Among 11,401 Methadone Patients With Convictions From BC, 2001-2015
