# Supplementary material for: Association of Methadone Treatment With Substance-Related Hospital Admissions Among a Population in Canada With a History of Criminal Convictions
Source: JAMA Netw Open. 2019 Mar 15;2(3):e190595. doi: 10.1001/jamanetworkopen.2019.0595 (PMC6484638; doi:10.1001/jamanetworkopen.2019.0595)
Supplement: Supplement. — eMethods 1. Description of Databases Used in the Study eTable 1. Summary of Information Available From the Ministry Of Health Database Used in the Current Study eTable 2. Summary of Information Available From the Ministry of Justice Database Used in the Current Study eMethods 2. Methods for Constructing Time Intervals and Time at Risk in the Cox Regression Model eTable 3. Illustration of Data Structure for the Primary Exposure Variable (Methadone) in the Cox Regression for Five Hypothetical Participants eTable 4. Illustration of Event Time (Admission & Discharge) for Five Hypothetical Participants eTable 5. Illustration of the Data Structure for the Primary Exposure and Outcome Variables in the Cox Regression for Five Hypothetical Participants eTable 6. Illustration of Summary Statistics for Five Hypothetical Participants eTable 7. Top 5 ICD-10-CA Diagnostic Categories Responsible for SUD, NSMD, and MED related acute hospitalizations among 11,401 Methadone Patients With Convictions From BC, 2001–2015 eTable 8. ICD-10 Diagnostic Codes (F10-F19) for SUD Related Acute Hospitalizations (n=3,205) eTable 9. ICD-10-CA Diagnostic Codes (F00-F09; F20-F99) for NSMD Related Acute Hospitalizations (n=2,232) eTable 10. ICD-10 Diagnostic Codes for MED Acute Hospitalizations (N = 13 723) eTable 11. ICD-9 Diagnostic CODES (290-319) for Mental Disorders eTable 12. Description of Methadone and Controlling Variables Included in the Multivariable Cox Regression eTable 13. Subgroup Analysis Estimating the Hazard Associated With Methadone for Acute Hospitalizations (Any Cause) Among 1,978 Methadone Patients With Convictions and Very Short Follow Up (<2 Years) From BC, 2001-2015 eTable 14. Subgroup Analysis Estimating the Hazard Associated With Methadone for Acute Hospitalizations (Any Cause) Among 3,248 Methadone Patients With Convictions and Short Follow Up (2-5 Years) From BC, 2001-2015 eTable 15. Subgroup Analysis Estimating the Hazard Associated With Methadone for Acute Hospitalizati [file jamanetwopen-2-e190595-s001.pdf]

Supplementary Online Content

Russolillo A, Moniruzzaman A, Somers JM. Association of methadone treatment with substance-related hospital admissions among a population in Canada with a history of criminal convictions. *JAMA Netw Open*. 2019;2(3):e190595. doi:10.1001/jamanetworkopen.2019.0595

eMethods 1. Description of Databases Used in the Study

eTable 1. Summary of Information Available From the Ministry Of Health Database Used in the Current Study

eTable 2. Summary of Information Available From the Ministry of Justice Database Used in the Current Study

eMethods 2. Methods for Constructing Time Intervals and Time at Risk in the Cox Regression Model

eTable 3. Illustration of Data Structure for the Primary Exposure Variable (Methadone) in the Cox Regression for Five Hypothetical Participants

eTable 4. Illustration of Event Time (Admission & Discharge) for Five Hypothetical Participants

eTable 5. Illustration of the Data Structure for the Primary Exposure and Outcome Variables in the Cox Regression for Five Hypothetical Participants

eTable 6. Illustration of Summary Statistics for Five Hypothetical Participants

eTable 7. Top 5 ICD-10-CA Diagnostic Categories Responsible for SUD, NSMD, and MED related acute hospitalizations among 11,401 Methadone Patients With Convictions From BC, 2001–2015

eTable 8. ICD-10 Diagnostic Codes (F10-F19) for SUD Related Acute Hospitalizations (n=3,205)

eTable 9. ICD-10-CA Diagnostic Codes (F00-F09; F20-F99) for NSMD Related Acute Hospitalizations (n=2,232)

eTable 10. ICD-10 Diagnostic Codes for MED Acute Hospitalizations (N=13,723)

eTable 11. ICD-9 Diagnostic CODES (290-319) for Mental Disorders

eTable 12. Description of Methadone and Controlling Variables Included in the Multivariable Cox Regression

eTable 13. Subgroup Analysis Estimating the Hazard Associated With Methadone for Acute Hospitalizations (Any Cause) Among 1,978 Methadone Patients With Convictions and Very Short Follow Up (< 2 Years) From BC, 2001-2015

eTable 14. Subgroup Analysis Estimating the Hazard Associated With Methadone for Acute Hospitalizations (Any Cause) Among 3,248 Methadone Patients With Convictions and Short Follow Up (2-5 Years) From BC, 2001-2015

eTable 15. Subgroup Analysis Estimating the Hazard Associated With Methadone for Acute Hospitalizations (Any Cause) Among 3,853 Methadone Patients With Convictions And Long Follow Up (5-10 Years) From BC, 2001-2015

eTable 16. Subgroup Analysis Estimating the Hazard Associated With Methadone for Acute Hospitalizations (Any Cause) Among 2,322 Methadone Patients With Convictions and Very Long Follow Up (>10 Years) From BC, 2001-2015

eTable 17. Subgroup Analysis Estimating the Hazard Associated With Methadone for Acute Hospitalizations (Any Cause) Among 10,430 Methadone Patients With Convictions Only Prescribed Methadone (No Buprenorphine or Buprenorphine-Naloxone) From BC, 2001-2015

eTable 18. Extended Cox Regression Analysis Estimating the Hazard Associated With Methadone for Cellulitis or Abscess Related Hospitalizations Among 11,401 Methadone Patients With Convictions From BC, 2001-2015

This supplementary material has been provided by the authors to give readers additional information about their work.

© 2019 Russolillo A et al. *JAMA Network Open.*

eMethods 1

Description of Databases Used in the Study

The Discharge Abstract Database (DAD) is a national (Canada) hospitalization database maintained by the Canadian Institute of Health information (CIHI). This registry captures administrative, clinical and demographic information on hospital discharges which includes detailed information related to hospital activities (such as admissions, discharges, transfers, length of stay, etc.), diagnoses information (associated with hospital separations), day-surgeries (in acute care). Select chronic care, rehabilitation, and psychiatric facilities also submit data to DAD.

All Canadian acute care facilities (except Quebec) are required to submit their discharge records directly to CIHI for inclusion in the DAD. The format of the DAD was changed significantly in 2001–2002, in response to some provinces and territories using ICD-10-CA and CCI to code diagnoses and interventions in hospital separations reported to the DAD. Since 2004–2005, all DAD records have been reported in ICD-10-CA and CCI; prior to that, ICD-9, CCP and ICD-9-CM were used. Each Canadian province receives their own version of DAD data which include several standardized CIHI variables (such as case mix groups, and resource intensity weights) in addition to the core DAD data elements. Detailed information about this database is available on the CIHI web site (www.cihi.ca). The centralized data processing of hospital separation records completed by the CIHI ensures standardization and access of data elements across the Canadian provinces.

The current study used the provincial version of the DAD, maintained by the British Columbia Ministry of Health. Information contained in this dataset includes: discharges, transfers and deaths of in-patients and day surgery patients from BC acute care hospitals. Although this provincial data captures information since 1990, this study used data from 2001 on wards to be consistent with the ICD-10-CA/CCI classification system changes. Moreover, this study also used several other databases maintained by the Ministry of Health and the Ministry of Justice. eTables 1 and 2 present a snapshot of information available from the Ministries of Health and Justice datasets used from the current study.

© 2019 Russolillo A et al. *JAMA Network Open.*

eTable 1 Summary of Information Available From the Ministry Of Health Database Used in the Current Study

Name of Database	**Ministry of Health: Available Information**	**Timeline**
**Medical Service Plan (MSP) billing data**	• Medical services delivered to patients covered by MSP<br>o Date, diagnostic code (ICD-9) and cost associated for each service (including laboratory and diagnostic procedures)<br>o Type of services	April 1990 to March 2015
**Discharge Abstract Database (DAD)**	• Activities including discharges, transfers and deaths for in-patient and day-surgery patients in BC Acute Care Hospitals<br>o Time of admission & discharge<br>o Length of stay in hospital<br>o Diagnostic codes and types<br>o Intervention/procedure codes<br>o Hospital codes<br>o Type of Disposition<br>o Mode of entry	April 1990 to March 2015
**PharmaCare and PharmaNet Data**	• Prescription service dates<br>• Drug codes/DIN, therapeutic codes<br>• Costs associated with ingredients and professional services<br>• Location of pharmacy<br>• Type of PharmaCare plan<br>• Type of drugs based on therapeutics class (such as, Opiate agonists, Antidepressants, Benzodiazepines, Tranquilizers),<br>• Type of drugs based American Hospital Formulary classification (such as, Antidepressants, Antipsychotic agents, Opiate agonists)<br>• Generic and brand name of drugs<br>• Quantity (e.g., 60 pills), dosage (e.g., 50 mg) and days of supply (e.g., 5 days)	April 1990 to March 2015
**Vital Statistics death file**	• Dates and causes of death (diagnostic codes)	April 1990 to March 2015

© 2019 Russolillo A et al. *JAMA Network Open.*

eTable 2Summary of Information Available From the Ministry of Justice Database Used in the Current Study

Name of Database	**Ministry of Justice: Available Information**	**Timeline**
**Sentence Database**	• Offences: dates, types and charge counts<br>• Sentences: dates, types and lengths of sentences<br>• Court that delivered sentences	April 1997 to March 2015
**Socio-demographic database**	• Gender<br>• Ethnicity<br>• Education level<br>• Birth date<br>• Age	April 1997 to March 2015

© 2019 Russolillo A et al. *JAMA Network Open.*

eMethods 2

Methods for Constructing Time Intervals and Time at Risk in the Cox Regression Model

In the current paper, we chose time-to-event/ survival analysis since our outcome of interest (hospital admission) was not only the occurrence of an event, but also when the event occurred. Moreover, censoring was another important consideration, in selecting time-to-event analysis. In the presence of censoring, exact time-to-event is undeterminable. Traditional regression analysis is not well equipped to incorporate both the occurrence of an event and time-to-event as an outcome. In addition, our analysis was complicated due to the recurrent nature of the outcome variable and the time-varying (values change over time) nature of the primary independent variable (methadone status: medicated vs. non-medicated period). Data set-up for survival analysis with time-varying covariates is an important consideration in conducting the Cox regression. The time-varying covariates can be incorporated in the time-to-event analysis by sub-dividing the follow-up time into intervals/segments. This data structure procedure is referred to as counting process (CP). This process essentially subdivides an individual's entire time at risk (follow-up time) into smaller time segments to absorb the change of values of the time-dependent covariates, resulting in multiple observations per participant.

Another important consideration in constructing the Cox regression model was understanding *actual time at risk*. Since our outcome was recurrent hospitalizations, once participants were admitted to hospital, they were not at risk for re-hospitalization until their discharge. To account for this phenomenon, time during hospitalization (hospital days) was excluded from the time at risk. This document is intended to illustrate how time intervals including actual time at risk for methadone were constructed and accounted for in the Cox regression model. We have chosen five hypothetical participants to describe this procedure. eTable 3 presents individual time intervals for methadone without considering the event of interest (outcome) and actual time at risk while eTable 4 only presents the event time. eTable 5 illustrates the framework of analytic data incorporating the outcome and time at risk.

Individual time intervals were calculated using pharmacy filing transaction dates. If a participant filled their methadone prescription consistently (no gap in pharmacy transactions dates) for a period of time, this was treated as a single interval/episode and considered as a medicated period (methadone was dispensed). If the participant didn't fill a prescription for a period of time (gap in pharmacy transaction dates), the interval was considered a non-medicated period (methadone was not dispensed). Participants were expected to alternate between medicated and non-medicated periods throughout follow-up.

© 2019 Russolillo A et al. *JAMA Network Open.*

eTable 3. Illustration of Data Structure for the Primary Exposure Variable (Methadone) in the Cox Regression for Five Hypothetical Participants

id	rx	sdt	edt	dur	time0	time1	n_int
A	1	01-Jan-14	31-Jan-14	30	0	30	1
A	0	31-Jan-14	28-Feb-14	28	30	58	2
A	1	28-Feb-14	28-May-14	89	58	147	3
A	0	28-May-14	30-May-14	2	147	149	4
A	1	30-May-14	30-Jun-14	31	149	180	5
A	0	30-Jun-14	01-Sep-14	63	180	243	6
A	1	01-Sep-14	30-Sep-14	29	243	272	7
A	0	30-Sep-14	30-Nov-14	61	272	333	8
A	1	30-Nov-14	30-Jan-15	61	333	394	9
A	0	30-Jan-15	08-Feb-15	9	394	403	10
A	1	08-Feb-15	28-Feb-15	20	403	423	11
A	0	28-Feb-15	31-Mar-15	31	423	454	12
B	1	10-Sep-14	23-Sep-14	13	0	13	1
B	0	23-Sep-14	24-Sep-14	1	13	14	2
B	1	24-Sep-14	13-Oct-14	19	14	33	3
B	0	13-Oct-14	14-Oct-14	1	33	34	4
B	1	14-Oct-14	20-Oct-14	6	34	40	5
B	0	20-Oct-14	21-Oct-14	1	40	41	6
B	1	21-Oct-14	22-Oct-14	1	41	42	7
B	0	22-Oct-14	24-Oct-14	2	42	44	8
B	1	24-Oct-14	29-Oct-14	5	44	49	9
B	0	29-Oct-14	31-Oct-14	2	49	51	10
B	1	31-Oct-14	10-Dec-14	40	51	91	11
C	1	15-Dec-12	31-Dec-12	16	0	16	1
C	0	31-Dec-12	24-Jan-13	24	16	40	2
C	1	24-Jan-13	16-May-13	112	40	152	3
C	0	16-May-13	19-Sep-13	126	152	278	4
C	1	19-Sep-13	19-Dec-13	91	278	369	5
C	0	19-Dec-13	02-Feb-14	45	369	414	6
C	1	02-Feb-14	05-Jun-14	123	414	537	7
C	0	05-Jun-14	02-Jan-15	211	537	748	8
C	1	02-Jan-15	02-Feb-15	31	748	779	9
C	0	02-Feb-15	03-Feb-15	1	779	780	10
C	1	03-Feb-15	18-Mar-15	43	780	823	11
C	0	18-Mar-15	31-Mar-15	13	823	836	12
D	1	15-Dec-12	15-Dec-13	365	0	365	1
E	1	16-Mar-12	16-Apr-12	31	0	31	1
E	0	16-Apr-12	25-Apr-12	9	31	40	2
E	1	25-Apr-12	25-Apr-13	365	40	405	3
E	0	25-Apr-13	25-Apr-14	365	405	770	4
E	1	25-Apr-14	25-Aug-14	122	770	892	5
E	0	25-Aug-14	25-Oct-14	61	892	953	6
E	1	31-Dec-14	31-Jan-15	31	953	984	7
E	0	31-Jan-15	31-Mar-15	59	984	1043	8

© 2019 Russolillo A et al. *JAMA Network Open.*

Id: identifier variable; rx: methadone (0-non-medicated status, 1-medicated status); sdt: start date of each time-interval; edt: end date of each time-interval; dur: duration of each time-interval; time0: start time of each time-interval, in days; time1: end time of each time-interval, in days; n_int: order of time interval

For example (eTable 3), participant A initiated methadone on Jan 1, 2014 and filled the methadone prescription continuously (no gap) until Jan 31, 2014 (this can be a single pharmacy transaction with 30 days of drug supply or 30 pharmacy transactions with a single day of drug supply for each transaction). Following Jan 31, 2014, participant A didn't have any pharmacy transactions until Feb 28, 2014. For this participant, January was considered a medicated time interval with a duration of 30 days and February was considered a non-medicated time interval with a duration of 28 days. After February, this participant received methadone for another 89 days (28-Feb-14 to 28-May-14) followed by two days off-treatment (28-May-14 to 30-May-14). This participant had a few more medicated and non-medicated episodes until censoring (study end, March 31, 2015). In total, participant A had twelve time-intervals: six medicated and six non-medicated.

However, participant A (eTable 4 & eTable 5) experienced an event (hospital admission) at 85 days of follow-up during a medicated time-interval (interval # 3) and was released from hospital at 89 days of follow up during the same medicated time-interval (interval # 3). Once participants were in hospital, they were not at risk for re-hospitalization until discharge. Participant A stayed in hospital for four days which was excluded from the follow-up time to estimate actual time at risk. To account for this phenomenon (event time & length of stay), interval # 3 (spanning 58 to 147 days) for participant A was split into three time time-span records (eTable 5): 58 to 85 days (to specify the event time which occurred at 85 days), 85 to 89 days (to specify the time span in hospital) and 89 to 147 days (to specify the end of interval # 3). The time-span 85 to 89 days was not considered as a time at risk (highlighted blue) and was excluded during the Cox regression analysis. Participant A had no further events until censoring. Total follow-up time for participant A was 454 days (Jan 1, 2014 to Mar 31, 2015) while time at risk was 450 days (follow-up time - length of stay in hospital). The overall event (hospitalization) rate (eTable 6) for this participant was 1/450 (0.8 per person-year). Since participant A experienced an event during a medicated time-interval, the event rate for medicated periods was 1/256 (1.4 per person-year) and for non-medicated periods was 0/194 (0.0 per person-year).

Participant B initiated methadone on 10-Sep-14 and was censored on 10-Dec-14 (before study end) due to death. This participant contributed only 91 analysis-days, mostly medicated time (84 days vs. 7 days) with 11 time- intervals. Since participant B didn't experience any events, the event (hospitalization) rate was zero for all instances (overall, medicated and non-medicated period).

© 2019 Russolillo A et al. *JAMA Network Open.*

eTable 4. Illustration of Event Time (Admission & Discharge) for Five Hypothetical Participants

id[1]	rx	time1	event
A	1	85	1
A	1	89	2
C	0	30	1
C	0	32	2
C	0	180	1
C	0	190	2
C	1	300	1
C	1	345	2
E	1	365	1
E	1	375	2
E	0	450	1
E	0	490	2
E	0	730	1
E	0	750	2

Id: identifier variable; time1: time of event, in days; event: 1-admitted to hospital (event occurred), 2-discharged from hospital

Participant C, had a similar profile to participant A, initiated methadone on 15-Dec-12 and was censored when the study ended (March 31, 2015). This participant had a long follow-up time of 836 days, but had only 12 time-intervals. Participant C is an example of multiple recurrences, because they experienced three events at 30, 180 and 300 days. The corresponding time in hospital for participant C was 2 (time span: 30 to 32 days), 10 (time span: 180 to 190 days) and 45 days (time span: 300 to 345 days). Participant C stayed in hospital for a total of 57 days and was at actual risk for 779 days (836 days - 57 days). The first two events happened during a non-medicated period and the last event happened during a medicated period. Therefore, the hospitalization rates for the entire period (including medicated and non-medicated periods), were 3/779 days (1.4 per person-year), 1/371 days (1.0 per person-year) & 2/408 days (1.8 per person-year) respectively.

[1]-Participant B & Participant D had no event of interest and were not shown in the table.

© 2019 Russolillo A et al. *JAMA Network Open.*

eTable 5 Illustration of the Data Structure for the Primary Exposure and Outcome Variables in the Cox Regression for Five Hypothetical Participants

id	rx	n_int	time0	time1	dur	event
A	1	1	0	30	30	0
A	0	2	30	58	28	0
A	1	3	58	85	27	1[2]
A	1	3	85	89	4	2[3]
A	1	3	89	147	58	0
A	0	4	147	149	2	0
A	1	5	149	180	31	0
A	0	6	180	243	63	0
A	1	7	243	272	29	0
A	0	8	272	333	61	0
A	1	9	333	394	61	0
A	0	10	394	403	9	0
A	1	11	403	423	20	0
A	0	12	423	454	31	0
B	1	1	0	13	13	0
B	0	2	13	14	1	0
B	1	3	14	33	19	0
B	0	4	33	34	1	0
B	1	5	34	40	6	0
B	0	6	40	41	1	0
B	1	7	41	42	1	0
B	0	8	42	44	2	0
B	1	9	44	49	5	0
B	0	10	49	51	2	0
B	1	11	51	91	40	0
C	1	1	0	16	16	0
C	0	2	16	30	14	1
C	0	2	30	32	2	2
C	0	2	32	40	8	0
C	1	3	40	152	112	0
C	0	4	152	180	28	1
C	0	4	180	190	10	2
C	0	4	190	278	88	0
C	1	5	278	300	22	1
C	1	5	300	345	45	2
C	1	5	345	369	24	0
C	0	6	369	414	45	0
C	1	7	414	537	123	0
C	0	8	537	748	211	0
C	1	9	748	779	31	0
C	0	10	779	780	1	0

[2]-Yellow highlights indicate a time span record when participants experienced an event (at time1, the end of time span).
[3]-Blue highlights indicate a time span record when participants were in hospital. This was not actual time at risk and was not included in the analysis.

© 2019 Russolillo A et al. *JAMA Network Open.*

id	rx	n_int	time0	time1	dur	event
C	1	11	780	823	43	0
C	0	12	823	836	13	0
D	1	1	0	365	365	0
E	1	1	0	31	31	0
E	0	2	31	40	9	0
E	1	3	40	365	325	1
E	1	3	365	375	10	2
E	1	3	375	405	30	0
E	0	4	405	450	45	1
E	0	4	450	490	40	2
E	0	4	490	730	240	1
E	0	4	730	750	20	2
E	0	4	750	770	20	0
E	1	5	770	892	122	0
E	0	6	892	953	61	0
E	1	7	953	984	31	0
E	0	8	984	1043	59	0

Id: identifier variable; rx: methadone (0-non-medicated status, 1-medicated status); n_int: order of time interval; time0: start time of each time-interval, in days; time1: end time of each time-interval, in days; dur: duration of each time-interval; event: 0 - no event, 1- admitted to hospital (event occurred), 2 - discharged from hospital

Participant D initiated methadone on 15-Dec-12 and was censored on 15-Dec-13 (before study end) due to death. This participant continued to receive methadone until censoring. Participant D didn't have any gaps in dispensed methadone, resulting in one medicated time-interval (no non-medicated time intervals) and a single observation. This participant was another example of a zero event (no hospital admissions). Therefore, the hospitalization rate was zero for all instances: overall (0/365 days, 0.0 per person-year), medicated period (0/365 days, 0.0 per person-year) and non-medicated period (0/0 days, 0.0 per person-year).

Participant E initiated methadone on 16-Mar-12 and was censored when the study ended (March 31, 2015). This participant had a long follow-up time of 1,043 days. This participant is another example of multiple recurrences, who experienced three events at 365, 450 and 730 days. The corresponding time in hospital for participant E was 10 (time span: 365 to 375 days), 40 (time span: 450 to 490 days) and 20 days (time span: 730 to 750 days). Participant E stayed in hospital for a total of 70 days and was at actual risk for 973 days (1043 days - 70 days). The first event happened during the medicated period and the last two events happened during the non-medicated periods. Therefore, the hospitalization rates for the entire period (including medicated and non-medicated periods), were 3/973 days (1.0 per person-year), 1/539 days (0.7 per person-year) & 2/434 days (1.4 per person-year) respectively.

© 2019 Russolillo A et al. *JAMA Network Open.*

eTable 6 Illustration of Summary Statistics for Five Hypothetical Participants

	**Participant A**	**Participant B**	**Participant C**	**Participant D**	**Participant E**	**Total[4]**
Censoring	Censored on March 31, 2015 (study end)	Censored on Dec 10, 2014 (before study end due to death)	Censored on March 31, 2015 (study end)	Censored on Dec 15, 2013 (before study end due to death)	Censored on March 31, 2015 (study end)	
# of time intervals	12	11	12	1	8	44
# of medicated intervals	6	6	6	1	4	23
# of non-medicated intervals	6	5	6	0	4	21
Total follow-up, days	454	91	836	365	1043	2789
Time spent in hospitals, days	4	0	57	0	70	131
Total time at risk, days	450	91	779	365	973	2658
Total medicated time at risk, days	256	84	371	365	539	1615
Total non-medicated time at risk, days	194	7	408	0	434	1043
Longest medicated interval, days	89	40	123	365	365	365
Longest non-medicated interval, days	63	2	211	0	365	365
Number of events (acute hospitalizations)	1	0	3	0	3	7
Event rate, per person-year<br>Overall<br>On Methadone (medicated period)<br>Off Methadone (non-medicated period)	 0.8<br>1.4<br>0.0	 0.0<br>0.0<br>0.0	 1.4<br>1.0<br>1.8	 0.0<br>0.0<br>0.0	 1.1<br>0.7<br>1.7	 1.0<br>0.7<br>1.4

[4]-Total was calculated using all five participants.

© 2019 Russolillo A et al. *JAMA Network Open.*

eTable 7. Top 5 ICD-10-CA Diagnostic Categories Responsible for SUD, NSMD, and MED related acute hospitalizations among 11,401 Methadone Patients With Convictions From BC, 2001–2015.

**SUD (n=3,205)**		
**ICD-10-CA CODE[5]**	**Description of disorder[6]**	**N (%)**
F19	Mental and behavioural disorders due to multiple drug use and use of other psychoactive substances	1,033 (32.3)[7]
F11	Mental and behavioural disorders due to use of opioids	701 (21.9)[8]
F10	Mental and behavioural disorders due to use of alcohol	525 (16.4)[9]
F14	Mental and behavioural disorders due to use of cocaine	473 (14.8)[10]
F15	Mental and behavioural disorders due to use of other stimulants, including caffeine	404 (12.6)[11]
**NSMD (n=2,232)**		
F29	Unspecified nonorganic psychosis	386 (17.3)
F32	Depressive episode	341 (15.3)
F20	Schizophrenia	315 (14.1)
F43	Reaction to severe stress, and adjustment disorders	256 (11.5)
F25	Schizoaffective disorders	194 (8.7)
**MED (n=13,723)[12]**		
L03	Cellulitis	905 (6.6)[13]
B24	Human immunodeficiency virus [HIV] disease	663 (4.8)
J18	Pneumonia, organism unspecified	539 (3.9)
J44	Other chronic obstructive pulmonary disease	486 (3.5)
T40	Poisoning by narcotics and psychodysleptics [hallucinogens]	395 (2.9)

BC: British Columbia; ICD: International Classification of Diseases, 10th Edition; NSMD: Non-Substance related Mental Disorder; MED: medical; SUD: Substance Use Disorder

[5] - Represents most responsible cause (primary) for in-hospital stay.
[6] - This table includes description of disorders as listed in the document: Canadian Institute for Health Information 2009, Volume 1, International Statistical Classification of Diseases and Related Health Problems, Tenth Revision, Canada (ICD-10-CA) - Tabular List
[7] -Among 1,033 admissions, the distribution by four-digit diagnostic codes were as follows: F190: 63 (6.1%); F191: 248 (24.0%); F192: 245 (23.7%); F193: 41 (4.0%); F194: 12 (1.2%); F195: 362 (35.0%); F196: < 5; F198: 56 (5.4%); F199: 5 (0.5%).
[8] - Among 701 admissions, the distribution by four-digit diagnostic codes were as follows: F110: 52 (7.4%); F111: 70 (10.0%); F112: 267 (38.1%); F113: 217 (31.0%); F114: 15 (2.1%); F115: 61 (8.7%); F118: 17 (2.4%); F118: 17 (2.4%); F119: < 5.
[9] - Among 525 admissions, the distribution by four-digit diagnostic codes was as follows: F100: 88 (16.8%); F101: 53 (10.1%); F102: 106 (20.2%); F103: 226 (43.1%); F104: 30 (5.7%); F105: 13 (2.5%); F106: < 5; F108: 7 (1.3%): F108: 56 (5.4%); F109: < 5.
[10] - Among 473 admissions, the distribution by four-digit diagnostic codes was as follows: F140: 48 (10.1%); F141: 104 (22.0%); F142: 144 (30.4%); F143: 30 (6.3%); F144: 5 (1.1%); F145: 127 (26.9%); F146: < 5; F148: 14 (3.0%).
[11] - Among 404 admissions, the distribution by four-digit diagnostic codes was as follows: F150: 45 (11.1%); F151: 43 (10.6%); F152: 39 (9.7%); F153: 9 (2.2%); F155: 260 (64.4%); F156: < 5; F158: < 5; F159: < 5.
[12] - The 505 (3.7%) admissions related to Other maternal diseases classifiable elsewhere but complicating pregnancy, childbirth, and the puerperium were excluded from the MED top 5 categories presented above
[13] - Among 905 admissions, the distribution by specific diagnostic codes was as follows: L0300: 20 (2.2%); L0301: < 5; L0310: 239 (26.4%); L0311: 553 (61.1%); L032: 34 (3.8%); L0330: < 5; L0331: < 5; L0333: < 5; L0334: < 5; L0335: < 5; L0336: < 5; L0339: < 5; L038: 19 (2.1%); L039: 10 (1.1%).

© 2019 Russolillo A et al. *JAMA Network Open.*

eTable 8. ICD-10 Diagnostic Codes (F10-F19) for SUD[14] Related Acute Hospitalizations (n=3,205)

ICD-10-CA CODE[15]	Description of disorder	N (%)
F10	Mental and behavioural disorders due to use of alcohol	525 (16.4)[16]
F11	Mental and behavioural disorders due to use of opioids	701 (21.9)[17]
F12	Mental and behavioural disorders due to use of cannabinoids	30 (1.0)
F13	Mental and behavioural disorders due to use of sedatives or hypnotics	25 (1.0)
F14	Mental and behavioural disorders due to use of cocaine	473 (14.8)[18]
F15	Mental and behavioural disorders due to use of other stimulants, including caffeine	404 (12.6)[19]
F16	Mental and behavioural disorders due to use of hallucinogens	14 (<1.0)
F19	Mental and behavioural disorders due to multiple drug use and use of other psychoactive substances	1,033 (32.3)[20]
	**Total**	**3,205 (100)**

ICD: International Classification of Diseases; SUD: Substance Use Disorder

[14] - This table includes description of disorders as listed in the document: Canadian Institute for Health Information 2009, Volume 1, International Statistical Classification of Diseases and Related Health Problems, Tenth Revision, Canada (ICD-10-CA) - Tabular List

[15] - Represents most responsible cause (primary) for in-hospital stay.

[16] - Among 525 admissions, the distribution by four-digit diagnostic codes was as follows: F100: 88 (16.8%); F101: 53 (10.1%); F102: 106 (20.2%); F103: 226 (43.1%); F104: 30 (5.7%); F105: 13 (2.5%); F106: < 5; F108: 7 (1.3%): F108: 56 (5.4%); F109: < 5.

[17] - Among 701 admissions, the distribution by four-digit diagnostic codes were as follows: F110: 52 (7.4%); F111: 70 (10.0%); F112: 267 (38.1%); F113: 217 (31.0%); F114: 15 (2.1%); F115: 61 (8.7%); F118: 17 (2.4%); F118: 17 (2.4%); F119: 2 (0.3%).

[18] - Among 473 admissions, the distribution by four-digit diagnostic codes was as follows: F140: 48 (10.1%); F141: 104 (22.0%); F142: 144 (30.4%); F143: 30 (6.3%); F144: 5 (1.1%); F145: 127 (26.9%); F146: < 5; F148: 14 (3.0%).

[19] - Among 404 admissions, the distribution by four-digit diagnostic codes was as follows: F150: 45 (11.1%); F151: 43 (10.6%); F152: 39 (9.7%); F153: 9 (2.2%); F155: 260 (64.4%); F156: < 5; F158: < 5; F159: < 5.

[20] -Among 1,033 admissions, the distribution by four-digit diagnostic codes were as follows: F190: 63 (6.1%); F191: 248 (24.0%); F192: 245 (23.7%); F193: 41 (4.0%); F194: 12 (1.2%); F195: 362 (35.0%); F196: < 5; F198: 56 (5.4%); F199: 5 (0.5%).

© 2019 Russolillo A et al. *JAMA Network Open.*

eTable 9. ICD-10-CA Diagnostic Codes (F00-F09; F20-F99) for NSMD[21] Related Acute Hospitalizations (n=2,232)

ICD-10-CA CODE[22]	Description of disorder	N (%)
F04	Organic amnesic syndrome, not induced by alcohol and other psychoactive substances	< 5[23]
F05	Delirium, not induced by alcohol and other psychoactive substances	30 (1.3)
F06	Other mental disorders due to brain damage and dysfunction and to physical disease	10 (0.5)
F07	Personality and behavioural disorders due to brain disease, damage and dysfunction	< 5
F09	Unspecified organic or symptomatic mental disorder	2 (0.1)
F20	Schizophrenia	315 (14.1)
F22	Persistent delusional disorders	36 (1.6)
F23	Acute and transient psychotic disorders	32 (1.4)
F24	Induced delusional disorder	< 5
F25	Schizoaffective disorders	194 (8.7)
F28	Other nonorganic psychotic disorders	< 5
F29	Unspecified nonorganic psychosis	386 (17.3)
F30	Manic episode	11 (0.5)
F31	Bipolar affective disorder	147 (6.6)
F32	Depressive episode	341 (15.3)
F33	Recurrent depressive disorder	88 (3.9)
F34	Persistent mood [affective] disorders	22 (1.0)
F38	Other mood [affective] disorders	5 (0.2)
F39	Unspecified mood [affective] disorder	76 (3.4)
F41	Other anxiety disorders	47 (2.1)
F42	Obsessive-compulsive disorder	< 5
F43	Reaction to severe stress, and adjustment disorders	256 (11.5)
F44	Dissociative [conversion] disorders	13 (0.6)

[21] -This table includes description of disorders as listed in the document: Canadian Institute for Health Information 2009, Volume 1, International Statistical Classification of Diseases and Related Health Problems, Tenth Revision, Canada (ICD-10-CA) - Tabular List.
[22] -Represents most responsible cause (primary) for in-hospital stay.
[23] -Cell values of 1 to 4 were suppressed and presented as < 5.

© 2019 Russolillo A et al. *JAMA Network Open.*

**ICD-10-CA CODE[22]**	**Description of disorder**	**N (%)**
F45	Somatoform disorders	6 (0.3)
F48	Other neurotic disorders	< 5
F50	Eating disorders	< 5
F53	Mental and behavioural disorders associated with the puerperium, not elsewhere classified	< 5
F55	Abuse of non-dependence-producing substances	< 5
F60	Specific personality disorders	144 (6.5)
F61	Mixed and other personality disorders	14 (0.6)
F63	Habit and impulse disorders	< 5
F64	Gender identity disorders	< 5
F65	Disorders of sexual preference	< 5
F68	Other disorders of adult personality and behaviour	8 (0.4)
F69	Unspecified disorder of adult personality and behaviour	< 5
F84	Pervasive developmental disorders	< 5
F90	Hyperkinetic disorders	< 5
F91	Conduct disorders	< 5
F92	Mixed disorders of conduct and emotions	18 (0.8)
F94	Disorders of social functioning with onset specific to childhood and adolescence	< 5
F99	Mental disorder, not otherwise specified	5 (0.2)
	**Total**	**2,232 (100)**

ICD: International Classification of Diseases; NSMD: Non-Substance related Mental Disorder

© 2019 Russolillo A et al. *JAMA Network Open.*

eTable 10. ICD-10 Diagnostic Codes for MED[24] Acute Hospitalizations (N=13,723)

ICD-10-CA CODE[25,26]	Description of disorder	N (%)
L03	Cellulitis	905 (6.6)[27]
B24	Human immunodeficiency virus [HIV] disease	663 (4.8)
J18	Pneumonia, organism unspecified	539 (3.9)
O99	Other maternal diseases classifiable elsewhere but complicating pregnancy, childbirth and the puerperium	505 (3.7)
J44	Other chronic obstructive pulmonary disease	486 (3.5)
T40[28]	Poisoning by narcotics and psychodysleptics [hallucinogens]	395 (2.9)
A41	Other sepsis	386 (2.8)
L02	Cutaneous abscess, furuncle and carbuncle	309 (2.3)[29]
R10	Abdominal and pelvic pain	277 (2.0)
I33	Acute and subacute endocarditis	240 (1.8)
M00	Pyogenic arthritis	183 (1.3)
M86	Osteomyelitis	173 (1.3)
S02	Fracture of skull and facial bones	157 (1.1)
E10	Type 1 diabetes mellitus	156 (1.1)
S82	Fracture of lower leg, including ankle	152 (1.1)
K85	Acute pancreatitis	143 (1.0)
M46	Other inflammatory spondylopathies	139 (1.0)

[24] - This table includes description of disorders as listed in the document: Canadian Institute for Health Information 2009, Volume 1, International Statistical Classification of Diseases and Related Health Problems, Tenth Revision, Canada (ICD-10-CA) - Tabular List.
[25] - Represents most responsible cause (primary) for in-hospital stay.
[26] -Presented as descending order of frequency (largest to smallest) of diagnostic codes.
[27] - Among 905 admissions, the distribution by specific diagnostic codes was as follows: L0300: 20 (2.2%); L0301: < 5; L0310: 239 (26.4%); L0311: 553 (61.1%); L032: 34 (3.8%); L0330: < 5; L0331: < 5; L0333: < 5; L0334: < 5; L0335: 12 (1.3%); L0336: < 5; L0339: < 5; L038: 19 (2.1%); L039: 10 (1.1%).
[28] - Among 395 admissions, the distribution by four-digit diagnostic codes was as follows: T400: < 5; T401: 100 (25.3%); T402: 44 (11.1%); T404: 6 (1.5%); T405: 86 (21.8%); T406: 37 (9.4%).
[29] - Among 309 admissions, the distribution by four-digit diagnostic codes was as follows: L020: 7 (2.3%); L021: 32 (10.4%); L022: 20 (6.5%); L023: 30 (9.7%); L024: 210 (68.0%); L028: 7 (2.3%); L029: < 5.

© 2019 Russolillo A et al. *JAMA Network Open.*

ICD-10-CA CODE[25,26]	Description of disorder	N (%)
T42[30]	Poisoning by antiepileptic, sedative-hypnotic and antiparkinsonism drugs	139 (1.0)
S06	Intracranial injury	136 (1.0)
T43[31]	Poisoning by psychotropic drugs, not elsewhere classified	132 (1.0)
J45	Asthma	119 (0.9)
R07	Pain in throat and chest	119 (0.9)
A49	Bacterial infection of unspecified site	112 (0.8)
Z51	Other medical care	111 (0.8)
Z50	Care involving use of rehabilitation procedures	108 (0.8)
T84	Complications of internal orthopaedic prosthetic devices, implants and grafts	105 (0.8)
T81	Complications of procedures, not elsewhere classified	98 (0.7)
K80	Cholelithiasis	89 (0.7)
S52	Fracture of forearm	88 (0.6)
N39	Other disorders of urinary system	82 (0.6)
R45	Symptoms and signs involving emotional state	81 (0.6)
N17	Acute renal failure	79 (0.6)
O68	Labour and delivery complicated by fetal stress [distress]	79 (0.6)
G40	Epilepsy	77 (0.6)
S27	Injury of other and unspecified intrathoracic organs	77 (0.6)
K56	Vascular disorders of intestine	75 (0.6)
R50	Fever of other and unknown origin	75 (0.6)
O60	Preterm labour and delivery	74 (0.5)
K92	Other diseases of digestive system	73 (0.5)
R11	Nausea and vomiting	73 (0.5)
R41	Other symptoms and signs involving cognitive functions and awareness	72 (0.5)
K72	Hepatic failure, not elsewhere classified	70 (0.5)

[30]-Among 139 admissions, the distribution by four-digit diagnostic codes was as follows: T420: < 5; T424: 111 (79.9%); T425: < 5; T426: 18 (13.0%); T427: < 5; T428: < 5.
[31]-Among 132 admissions, the distribution by four-digit diagnostic codes was as follows: T430: 21 (15.9%); T432: 25 (18.9%); T433: < 5; T434: < 5; T435: 31 (23.5%); T436: 50 (37.9%); T439: < 5.

© 2019 Russolillo A et al. *JAMA Network Open.*

ICD-10-CA CODE[25,26]	Description of disorder	N (%)
M54	Dorsalgia	70 (0.5)
O34	Maternal care for known or suspected abnormality of pelvic organs	70 (0.5)
A40	Streptococcal sepsis	68 (0.5)
S72	Fracture of femur	67 (0.5)
E11	Type 2 diabetes mellitus	66 (0.5)
J69	Pneumonitis due to solids and liquids	62 (0.5)
K70	Alcoholic liver disease	61 (0.4)
M62	Other disorders of muscle	61 (0.4)
K35	Acute appendicitis	59 (0.4)
R56	Convulsions, not elsewhere classified	58 (0.4)
G06	Intracranial and intraspinal abscess and granuloma	54 (0.4)
S36	Injury of intra-abdominal organs	52 (0.4)
I21	Acute myocardial infarction	51 (0.4)
J15	Bacterial pneumonia, not elsewhere classified	51 (0.4)
J86	Pyothorax	50 (0.4)
S32	Fracture of lumbar spine and pelvis	50 (0.4)
A09	Other gastroenteritis and colitis of infectious and unspecified origin	49 (0.4)
O62	Abnormalities of forces of labour	48 (0.4)
E87	Other disorders of fluid, electrolyte and acid-base balance	47 (0.3)
Z54	Convalescence	47 (0.3)
L97	Ulcer of lower limb, not elsewhere classified	44 (0.3)
T39	Poisoning by nonopioid analgesics, antipyretics and antirheumatics	44 (0.3)
T82	Complications of cardiac and vascular prosthetic devices, implants and grafts	44 (0.3)
K29	Gastritis and duodenitis	43 (0.3)
S42	Fracture of shoulder and upper arm	42 (0.3)
I26	Pulmonary embolism	39 (0.3)

© 2019 Russolillo A et al. *JAMA Network Open.*

ICD-10-CA CODE[25,26]	Description of disorder	N (%)
J96	Respiratory failure, not elsewhere classified	39 (0.3)
M84	Disorders of continuity of bone	39 (0.3)
O42	Premature rupture of membranes	39 (0.3)
S22	Fracture of rib(s), sternum and thoracic spine	39 (0.3)
I50	Heart failure	37 (0.3)
K43	Ventral hernia	37 (0.3)
O70	Perineal laceration during delivery	37 (0.3)
K50	Crohn's disease [regional enteritis]	35 (0.3)
K52	Other noninfective gastroenteritis and colitis	35 (0.3)
M51	Other intervertebral disc disorders	35 (0.3)
L08	Other local infections of skin and subcutaneous tissue	34 (0.3)
M79	Other soft tissue disorders, not elsewhere classified	34 (0.3)
K74	Fibrosis and cirrhosis of liver	33 (0.2)
J13	Pneumonia due to Streptococcus pneumoniae	32 (0.2)
K59	Other functional intestinal disorders	32 (0.2)
N20	Calculus of kidney and ureter	32 (0.2)
S01	Open wound of head	32 (0.2)
S92	Fracture of foot, except ankle	32 (0.2)
M17	Gonarthrosis [arthrosis of knee]	31 (0.2)
M25	Other joint disorders, not elsewhere classified	31 (0.2)
N12	Tubulo-interstitial nephritis, not specified as acute or chronic	30 (0.2)
O03	Spontaneous abortion	30 (0.2)
M16	Coxarthrosis [arthrosis of hip]	29 (0.2)
M60	Myositis	29 (0.2)
M65	Synovitis and tenosynovitis	29 (0.2)
N73	Other female pelvic inflammatory diseases	29 (0.2)

© 2019 Russolillo A et al. *JAMA Network Open.*

ICD-10-CA CODE[25,26]	Description of disorder	N (%)
O64	Obstructed labour due to malposition and malpresentation of fetus	29 (0.2)
T50	Poisoning by diuretics and other and unspecified drugs, medicaments and biological substances	29 (0.2)
Z76	Persons encountering health services in other circumstances	29 (0.2)
A04	Other bacterial intestinal infections	28 (0.2)
O36	Maternal care for other known or suspected fetal problems	28 (0.2)
I80	Phlebitis and thrombophlebitis	27 (0.2)
O47	False labour	26 (0.2)
N13	Obstructive and reflux uropathy	25 (0.2)
R52	Pain, not elsewhere classified	25 (0.2)
Other[32]		3,058 (22.3)
	**Total**	**13,723 (100)**

ICD: International Classification of Diseases; MED: medical

[32] -Diagnostic codes with a frequency < 25 were not presented in the table and collapsed to a single category as other.

© 2019 Russolillo A et al. *JAMA Network Open.*

eTable 11.ICD-9 Diagnostic CODES (290-319) for Mental Disorders[33]

ICD-9 CODE	Description of disorder
290	SENILE AND PRESENILE ORGANIC PSYCHOTIC CONDITIONS
291*	*ALCOHOLIC PSYCHOSES*
292*	*DRUG PSYCHOSES*
293	TRANSIENT ORGANIC PSYCHOTIC CONDITIONS
294	OTHER ORGANIC PSYCHOTIC CONDITIONS (CHRONIC)
295	SCHIZOPHRENIC PSYCHOSES
296	AFFECTIVE PSYCHOSES
297	PARANOID STATES
298	OTHER NONORGANIC PSYCHOSES
299	PSYCHOSES WITH ORIGIN SPECIFIC TO CHILDHOOD
300	NEUROTIC DISORDERS
301	PERSONALITY DISORDERS
302	SEXUAL DEVIATIONS AND DISORDERS
303*	*ALCOHOL DEPENDENCE SYNDROME*
304*	*DRUG DEPENDENCE*
305*	*NONDEPENDENT ABUSE OF DRUGS*
306	PHYSIOLOGICAL MALFUNCTION ARISING FROM MENTAL FACTORS
307	SPECIAL SYMPTOMS OR SYNDROMES NOT ELSEWHERE CLASSIFIED
308	ACUTE REACTION TO STRESS
309	ADJUSTMENT REACTION

[33] -This table includes description of disorders as listed in the document accessed on May 18, 2018. URL: http://www2.gov.bc.ca/assets/gov/health/practitioner-pro/medical-services-plan/diag-codes_mental.pdf

© 2019 Russolillo A et al. *JAMA Network Open.*

ICD-9 CODE	Description of disorder
310	SPECIFIC NONPSYCHOTIC MENTAL DISORDERS FOLLOWING ORGANIC BRAIN DAMAGE
311	DEPRESSIVE DISORDER, NOT ELSEWHERE CLASSIFIED
312	DISTURBANCE OF CONDUCT NOT ELSEWHERE CLASSIFIED
313	DISTURBANCE OF EMOTIONS SPECIFIC TO CHILDHOOD AND ADOLESCENCE
314	HYPERKINETIC SYNDROME OF CHILDHOOD
315	SPECIFIC DELAYS IN DEVELOPMENT
316	PSYCHIC FACTORS ASSOCIATED WITH DISEASES CLASSIFIED ELSEWHERE
317	MILD MENTAL RETARDATION
318	OTHER SPECIFIED MENTAL RETARDATION
319	UNSPECIFIED MENTAL RETARDATION
50B	ANXIETY/DEPRESSION

ICD: International Classification of Diseases;
* Indicates the diagnostic codes used to ascertain Substance Use Disorders (SUD).

© 2019 Russolillo A et al. *JAMA Network Open.*

eTable `12. Description of Methadone and Controlling Variables Included in the Multivariable Cox Regression

Name of Variables	**Time of measurement**	**Analytic type & levels**	**Time-varying**	**Reference level**
Methadone	During the follow up period (Initiation to death or study end, march 31, 2015)	Binary (medicated & non-medicated)	Yes	Non-medicated period
Age	At the time of methadone initiation	Categorical with five levels<br><25 years, 25 to 34 years, 35 to 44 years, 45 to 54 years & 55 years or older	No	<25 years
Gender	Self-reported, time of justice contact	Binary (men & women)	No	Women
Ethnicity	Self-reported, time of justice contact	Categorical with three levels<br>White, Indigenous & Other<br>Unknown included as separate level	No	Other
Education level	Self-reported, time of justice contact	Categorical with four levels<br><Grade 10, Grade 10/11, Grade 12 & Vocational /University<br>Unknown included as separate level	No	Vocational /University
Year of methadone initiation	Calendar year of methadone initiation	Categorical with three levels<br>2001 to 2005, 2006 to 2010, 2011 to 2015[34]	No	2001 to 2005
Any offence	In the one-year period prior to methadone initiation	Categorical with three levels<br>None, 1-2 offences & > 2 offences	No	No offence
Severe mental illness	Ever	Categorical with three levels<br>No Schizophrenia/Bipolar, Schizophrenia[35] & Bipolar	No	No Schizophrenia/Bipolar
MSP services (SUD[36] related)	In the one-year period prior to methadone initiation	Categorical with three levels<br>Low[37] (≤ 1), Medium (2 to 6) & High (≥7)	No	Low (≤ 1)
MSP cost	In the one-year period prior to methadone initiation	Categorical with four levels[38]<br>1st, 2nd , 3rd and 4th quartile	No	1st quartile

[34] -2015 included only three months (January to March) of data
[35] -Three digits ICD-9 code 295 and 296 were used to ascertain Schizophrenia and Bipolar respectively. A hierarchical approach (schizophrenia first, then bipolar) was followed to distinguish between schizophrenia and bipolar diagnoses among participants who meet the criteria for both disorders.
[36]- Substance use disorders (SUD) were identified using the three-digit codes of 291, 292, 303, 304, and 305.
[37] -50th & 75th percentile was used to categorize into low, medium and high groups
[38] -25th, 50th & 75th percentile was used to categorize into four quartiles.

© 2019 Russolillo A et al. *JAMA Network Open.*

Name of Variables	**Time of measurement**	**Analytic type & levels**	**Time-varying**	**Reference level**
History of hospitalizations	In the one-year period prior to methadone initiation	Binary (No vs. Yes)	No	No

MSP: Medical Service Plan; SUD: Substance Use Disorder

eTable 13. Subgroup Analysis Estimating the Hazard Associated With Methadone for Acute Hospitalizations (Any Cause) Among 1,978[39] Methadone Patients With Convictions and Very Short Follow Up (< 2 Years) From BC, 2001-2015

Time Segment (years)	Medicated Methadone Period	Total Admissions	Total PYs	Incidence per 100 PYs	UHR (95% CI)[40]	AHR[41] (95% CI)	Risk difference[42] per 100 PYs (95% CI)
≤2.0	No	608	920.4	66.1	Reference	Reference	
	Yes	338	953.8	35.4	**0.51 (0.41, 0.64)**	**0.52 (0.42, 0.65)**	**-31.7 (-38.3, -23.1)**
Overall	Total	946	1,874.1	50.5	Reference	Reference	

AHR: Adjusted Hazard Ratio; BC: British Columbia; CI: Confidence Interval; PYs: Person-Years; UHR: Unadjusted Hazard Ratio

[39] - Analysis was restricted to participants who had less than 2 years (≤730 days) follow-up.
[40] -95% CIs and both unadjusted and adjusted hazard ratios were estimated using Robust Standard Errors.
[41] -The multivariable Cox model was controlled for age at enrolment (18 < 25 years, 25 < 35 years, 35 < 45 years, 45 < 55 years, and ≥ 55); gender (men & women); ethnicity (White, Indigenous, Other & Unknown); education level (<Grade 10, Grade 10/11, Grade 12, Vocational/University & Unknown); calendar year (2001 to 2005, 2006 to 2010 & 2011 to 2015), offences in the previous year (none, 1-2 & ≥3 offences), MSP cost in the previous year (quartile variable), SUD related services in the previous year (0-1, 2-6 & ≥7 services), severe mental illness (No Schizophrenia or Bipolar, Schizophrenia & Bipolar) & hospitalizations in the previous year (no vs. yes).
[42] - Risk difference was calculated using the formula: $I_0 \times (HR_a - 1)$, where I_0 indicates unadjusted event rate (incidence) in the methadone-no group and HR_a indicates the adjusted HR.

© 2019 Russolillo A et al. *JAMA Network Open.*

eTable 14. Subgroup Analysis Estimating the Hazard Associated With Methadone for Acute Hospitalizations (Any Cause) Among 3,248[43] Methadone Patients With Convictions and Short Follow Up (2-5 Years) From BC, 2001-2015

Time Segment (years)	Medicated Methadone Period	Total Admissions	Total PYs	Incidence per 100 PYs	UHR (95% CI) [44]	AHR[45] (95% CI)	Risk difference[46] per 100 PYs (95% CI)
≤2.0	No	1,328	3,156.4	42.1	Reference	Reference	
	Yes	666	3,298.6	20.2	**0.47 (0.41, 0.54)**	**0.46 (0.41, 0.53)**	**-22.7 (-24.8, -19.8)**
2.1 to ≤5.0	No	1,245	3,027.9	41.1	Reference	Reference	
	Yes	461	1,812.0	25.4	**0.62 (0.53, 0.73)**	**0.57 (0.48, 0.66)**	**-17.7 (-21.4, -14.0)**
Overall	No	2,573	6,184.3	41.6			
	Yes	1,127	5,110.6	22.1			
	Total	3,700	1,1294.9	32.8			

AHR: Adjusted Hazard Ratio; BC: British Columbia; CI: Confidence Interval; PYs: Person-Years; UHR: Unadjusted Hazard Ratio

[43] - Analysis was restricted to participants who had 2-5 years (731-1826 days) follow-up.
[44] -95% CIs and both unadjusted and adjusted hazard ratios were estimated using Robust Standard Errors.
[45] -The multivariable Cox model was controlled for age at enrolment (18 < 25 years, 25 < 35 years, 35 < 45 years, 45 < 55 years, and ≥ 55); gender (men & women); ethnicity (White, Indigenous, Other & Unknown); education level (<Grade 10, Grade 10/11, Grade 12, Vocational/University & Unknown); calendar year (2001 to 2005, 2006 to 2010 & 2011 to 2015), offences in the previous year (none, 1-2 & ≥3 offences), MSP cost in the previous year (quartile variable), SUD related services in the previous year (0-1, 2-6 & ≥7 services), severe mental illness (No Schizophrenia or Bipolar, Schizophrenia & Bipolar) & hospitalizations in the previous year (no vs. yes).
[46] - Risk difference was calculated using the formula: $I_0 \times (HR_a - 1)$, where I_0 indicates unadjusted event rate (incidence) in the methadone-no group and HR_a indicates the adjusted HR.

© 2019 Russolillo A et al. *JAMA Network Open.*

eTable 15. Subgroup Analysis Estimating the Hazard Associated With Methadone for Acute Hospitalizations (Any Cause) Among 3,853[47] Methadone Patients With Convictions And Long Follow Up (5-10 Years) From BC, 2001-2015

Time Segment (years)	Medicated Methadone Period	Total Admissions	Total PYs	Incidence per 100 PYs	UHR (95% CI)[48]	AHR[49] (95% CI)	Risk difference[50] per 100 PYs (95% CI)
≤2.0	No	1,434	3,583.2	40.0	Reference	Reference	
	Yes	979	4,068.4	24.1	**0.56 (0.50, 0.63)**	**0.53 (0.47, 0.59)**	**-18.8 (-21.2, -16.4)**
2.1 to ≤5.0	No	1,947	6,505.9	29.9	Reference	Reference	
	Yes	1,107	4,981.5	22.2	**0.74 (0.66, 0.83)**	**0.68 (0.61, 0.77)**	**-9.6 (-11.7, -6.9)**
5.1 to ≤10.0	No	1,630	5,014.6	32.5	Reference	Reference	
	Yes	873	3,346.3	26.1	**0.80 (0.70, 0.92)**	**0.75 (0.66, 0.86)**	**-8.1 (-11.1, -4.6)**
Overall	No	5,011	15,103.7	33.2			
	Yes	2,959	12,396.2	23.9			
	Total	7,970	27,499.9	29.0			

AHR: Adjusted Hazard Ratio; BC: British Columbia; CI: Confidence Interval; PYs: Person-Years; UHR: Unadjusted Hazard Ratio

[47] - Analysis was restricted to participants who had 5-10 years (1827 to 3652 days) follow-up.
[48] -95% CIs and both unadjusted and adjusted hazard ratios were estimated using Robust Standard Errors.
[49] -The multivariable Cox model was controlled for age at enrolment (18 < 25 years, 25 < 35 years, 35 < 45 years, 45 < 55 years, and ≥ 55); gender (men & women); ethnicity (White, Indigenous, Other & Unknown); education level (<Grade 10, Grade 10/11, Grade 12, Vocational/University & Unknown); calendar year (2001 to 2005, 2006 to 2010 & 2011 to 2015), offences in the previous year (none, 1-2 & ≥3 offences), MSP cost in the previous year (quartile variable), SUD related services in the previous year (0-1, 2-6 & ≥7 services), severe mental illness (No Schizophrenia or Bipolar, Schizophrenia & Bipolar) & hospitalizations in the previous year (no vs. yes).
[50] - Risk difference was calculated using the formula: $I_0 \times (HR_a - 1)$, where I_0 indicates unadjusted event rate (incidence) in the methadone-no group and HR_a indicates the adjusted HR.

© 2019 Russolillo A et al. *JAMA Network Open.*

eTable 16. Subgroup Analysis Estimating the Hazard Associated With Methadone for Acute Hospitalizations (Any Cause) Among 2,322[51] Methadone Patients With Convictions and Very Long Follow Up (>10 Years) From BC, 2001-2015

Time Segment (years)	Medicated Methadone Period	Total Admissions	Total PYs	Incidence per 100 PYs	UHR (95% CI) [52]	AHR[53] (95% CI)	Risk difference[54] per 100 PYs (95% CI)
≤2.0	No	759	2324.5	32.7	Reference	Reference	
	Yes	433	2291.7	18.9	**0.56 (0.48, 0.66)**	**0.53 (0.45, 0.61)**	**-15.3 (-18.0, -12.7)**
2.1 to ≤5.0	No	1293	4509.8	28.7	Reference	Reference	
	Yes	489	2413.1	20.3	**0.71 (0.61, 0.82)**	**0.63 (0.54, 0.74)**	**-10.6 (-13.2, -7.5)**
5.1 to ≤10.0	No	586	7006.0	22.8	Reference	Reference	
	Yes	873	4543.2	19.2	**0.84 (0.73, 0.98)**	**0.75 (0.65, 0.87)**	**-5.7 (-8.0, -3.0)**
>10.0	No	646	3101.8	20.8	Reference	Reference	
	Yes	453	1960.7	23.1	1.11 (0.92, 1.34)	0.99 (0.82 1.20)	-0.2 (-3.7, 4.2)
Overall	No	4296	16942.1	25.4			
	Yes	2248	11208.7	20.1			
	Total	6544	28150.8	23.2			

AHR: Adjusted Hazard Ratio; BC: British Columbia; CI: Confidence Interval; PYs: Person-Years; UHR: Unadjusted Hazard Ratio

[51] - Analysis was restricted to participants who had over 10 years (≥ 3653 days) of follow-up.
[52] -95% CIs and both unadjusted and adjusted hazard ratios were estimated using Robust Standard Errors.
[53] -The multivariable Cox model was controlled for age at enrolment (18 < 25 years, 25 < 35 years, 35 < 45 years, 45 < 55 years, and ≥ 55); gender (men & women); ethnicity (White, Indigenous, Other & Unknown); education level (<Grade 10, Grade 10/11, Grade 12, Vocational/University & Unknown); calendar year (2001 to 2005, 2006 to 2010 & 2011 to 2015), offences in the previous year (none, 1-2 & ≥3 offences), MSP cost in the previous year (quartile variable), SUD related services in the previous year (0-1, 2-6 & ≥7 services), severe mental illness (No Schizophrenia or Bipolar, Schizophrenia & Bipolar) & hospitalizations in the previous year (no vs. yes).
[54] - Risk difference was calculated using the formula: $I_0 \times (HR_a - 1)$, where I_0 indicates unadjusted event rate (incidence) in the methadone-no group and HR_a indicates the adjusted HR.

© 2019 Russolillo A et al. *JAMA Network Open.*

eTable 17. Subgroup Analysis Estimating the Hazard Associated With Methadone for Acute Hospitalizations (Any Cause) Among 10,430[55] Methadone Patients With Convictions Only Prescribed Methadone (No Buprenorphine or Buprenorphine-Naloxone) From BC, 2001-2015

Time Segment[56] (years)	Medicated Methadone Period	Total Admissions	Total PYs	Incidence per 100 PYs	UHR[57] (95% CI)[58]	AHR[59] (95% CI)	Risk difference[60] per 100 PYs (95% CI)
≤2.0	No	3,631	8,979.1	40.4	Reference	Reference	
	Yes	2,205	9,787.2	22.5	**0.52 (0.48, 0.57)**	**0.49 (0.46, 0.53)**	**-20.6 (-21.8, -19.0)**
2.1 to ≤5.0	No	3,986	12,760.9	31.2	Reference	Reference	
	Yes	1,907	8,510.1	22.4	**0.72 (0.66, 0.78)**	**0.64 (0.59, 0.70)**	**-11.2 (-12.8, -9.4)**
5.1 to ≤10.0	No	2,899	11,140.0	26.0	Reference	Reference	
	Yes	1,612	7,342.1	22.0	**0.84 (0.76, 0.94)**	**0.76 (0.69, 0.85)**	**-6.2 (-8.1, -3.9)**
>10.0	No	542	2,910.1	18.6	Reference	Reference	
	Yes	413	1,850.8	22.3	1.20 (0.97, 1.47)	1.07 (0.87, 1.31)	1.3 (-2.4, 5.8)
Overall	No	11,058	35,790.1	30.9			
	Yes	6,137	27,490.2	22.3			
	Total	17,195	63,280.3	27.2			

AHR: Adjusted Hazard Ratio; BC: British Columbia; CI: Confidence Interval; PYs: Person-Years; UHR: Unadjusted Hazard Ratio

[55] - Analysis was restricted to methadone recipient participants who didn't receive buprenorphine or buprenorphine-naloxone during the study period (n=10,430). Only 971 participants (8.5%) were prescribed buprenorphine or buprenorphine-naloxone as well as methadone during the study period.
[56] -The range of days for the four time-segments (≤2.0 years, 2.0 to ≤ 5.0 years, 5.0 to ≤10.0 years & > 10.0 years) are as follows: ≤ 730 days; 731 to 1826 days; 1827 to 3652 days & 3653 to 5111 days.
[57] -This Cox model includes treatment and the interaction terms with time-segments (at 2, 5 & 10 years)
[58] -95% CIs and both unadjusted and adjusted hazard ratios were estimated using Robust Standard Errors.
[59] -The multivariable Cox model was controlled for age at enrolment (18 < 25 years, 25 < 35 years, 35 < 45 years, 45 < 55 years, and ≥ 55); gender (men & women); ethnicity (White, Indigenous, Other & Unknown); education level (<Grade 10, Grade 10/11, Grade 12, Vocational/University & Unknown); calendar year (2001 to 2005, 2006 to 2010 & 2011 to 2015), offences in the previous year (none, 1-2 & ≥3 offences), MSP cost in the previous year (quartile variable), SUD related services in the previous year (0-1, 2-6 & ≥7 services), severe mental illness (No Schizophrenia or Bipolar, Schizophrenia & Bipolar) & hospitalizations in the previous year (no vs. yes).
[60] - Risk difference was calculated using the formula: $I_0 \times (HR_a - 1)$, where I_0 indicates unadjusted event rate (incidence) in the methadone-no group and HR_a indicates the adjusted HR.

© 2019 Russolillo A et al. *JAMA Network Open.*

eTable 18. Extended Cox Regression Analysis Estimating the Hazard Associated With Methadone for Cellulitis[61] or Abscess Related Hospitalizations Among 11,401 Methadone Patients With Convictions From BC, 2001-2015

Time Segment (years)	Medicated Methadone Period	Cellulitis or abscess related admissions[62]	Total PYs	Incidence per 100 PYs	UHR (95% CI)[63]	AHR[64] (95% CI)	Risk difference[65] per 100 PYs (95% CI)
≤2.0	No	316	9984.5	3.2	Reference	Reference	
	Yes	118	10612.5	1.1	**0.34 (0.26, 0.44)**	**0.33 (0.25, 0.43)**	**-2.1 (-2.4, -1.8)**
2.1 to ≤5.0	No	350	14043.6	2.5	Reference	Reference	
	Yes	109	9206.6	1.2	**0.47 (0.37, 0.61)**	**0.45 (0.35, 0.58)**	**-1.4 (-1.6, -1.0)**
>5.0[66]	No	194	15122.4	1.3	Reference	Reference	
	Yes	127	9850.1	1.3	1.01 (0.74, 1.37)	0.93 (0.68 1.26)	-0.1 (-0.4, 0.3)
Overall	No	860	39150.5	2.2			
	Yes	350	29669.3	1.2			
	Total	1214	68819.8	1.8			

AHR: Adjusted Hazard Ratio; BC: British Columbia; CI: Confidence Interval; PYs: Person-Years; UHR: Unadjusted Hazard Ratio

[61] -Three-digit ICD-10 codes (most responsible cause for in-hospital stay) were used to determine cellulitis (L03) or abscess (L02) related hospitalizations.
[62] -Out of 1,214 admissions during the study period, 905 were related to cellulitis (L03) and 309 were related to abscess (L02). 741 participants (6.5%) had at least one cellulitis or abscess related hospitalizations.
[63] -95% CIs and both unadjusted and adjusted hazard ratios were estimated using Robust Standard Errors.
[64] -The multivariable Cox model was controlled for age at enrolment (18 < 25 years, 25 < 35 years, 35 < 45 years, 45 < 55 years, and ≥ 55); gender (men & women); ethnicity (White, Indigenous, Other & Unknown); education level (<Grade 10, Grade 10/11, Grade 12, Vocational/University & Unknown); calendar year (2001 to 2005, 2006 to 2010 & 2011 to 2015), offences in the previous year (none, 1-2 & ≥3 offences), MSP cost in the previous year (quartile variable), SUD related services in the previous year (0-1, 2-6 & ≥7 services), severe mental illness (No Schizophrenia or Bipolar, Schizophrenia & Bipolar) & hospitalizations in the previous year (no vs. yes).
[65] - Risk difference was calculated using the formula: $I_0 \times (HR_a - 1)$, where I_0 indicates unadjusted event rate (incidence) in the methadone-no group and HR_a indicates the adjusted HR.
[66] -There were few admissions (45) in over 10-years group. The time segment (5-10 years) and over 10-years were collapsed to one group.

© 2019 Russolillo A et al. *JAMA Network Open.*
